# Punctal and Intracanalicular Drug Delivery Systems for Ophthalmic Use: A Narrative Review of Technologies, Clinical Outcomes, and Critical Quality Attributes

**DOI:** 10.3390/pharmaceutics18070830

**Published:** 2026-07-07

**Authors:** Elena O. Bakhrushina, Kseniia S. Leonova, Nikita O. Belyavsky, Vladimir I. Gegechkori, Vasily V. Belyaev, Boris B. Sysuev, Damir K. Salakhetdinov, Ivan I. Krasnyuk, Eugenia L. Atkova, Vasily D. Yartsev

**Affiliations:** 1 Department of Pharmaceutical Technology, A.P. Nelyubin Institute of Pharmacy, I.M. Sechenov First Moscow State Medical University, Moscow 119048, Russia; bakhrushina_e_o@staff.sechenov.ru (E.O.B.); belyavskiy_n_o@staff.sechenov.ru (N.O.B.); krasnyuk_i_i@staff.sechenov.ru (I.I.K.); 2 Department of Pharmaceutical and Toxicological Chemistry, A.P. Nelyubin Institute of Pharmacy, I.M. Sechenov First Moscow State Medical University, Moscow 119048, Russia; gegechkori_v_i@staff.sechenov.ru; 3 Department of Industrial Pharmacy, I.M. Sechenov First Moscow State Medical University, Moscow 119048, Russia; belyaev_v_v_1@staff.sechenov.ru; 4 Faculty of Bioengineering and Bioinformatics, M.V. Lomonosov Moscow State University, Moscow 119991, Russia; bsb500@yandex.ru; 5 Eurasian Academy of Good Practices (ANO “UANP”), Ext. Ter., Yakimanka Municipal District, Leninsky Ave., 9, Moscow 119049, Russia; 6 LLC “Chemical Diversity Research Institute”, Khimki 141401, Russia; dsalakhetdinov@yandex.ru; 7 Federal State Budgetary Institution of Science “M.M. Krasnov Research Institute of Eye Diseases” 11A, Rossolimo Street, Moscow 119021, Russia; evg.atkova@mail.ru (E.L.A.); v.yartsev@niigb.ru (V.D.Y.)

**Keywords:** punctal plug, intracanalicular delivery, sustained release, dexamethasone, lacrimal occlusion, quality by design (QbD), in situ gelation, canaliculitis

## Abstract

**Background:** Conventional ophthalmic eye drops have low bioavailability (<5%) and poor patient adherence, driving the development of sustained-release ophthalmic drug delivery systems. The lacrimal drainage system represents a unique anatomical site for minimally invasive depot formulations. **Objective:** To summarize and critically appraise punctal and intracanalicular drug delivery systems, occlusive devices, and in situ-forming hydrogels with respect to composition, release mechanisms, clinical efficacy, safety, and critical quality attributes (CQAs). **Methods:** A narrative literature review was conducted using PubMed, Scopus, Web of Science, Google Scholar, ClinicalTrials.gov, and patent/regulatory sources, including FDA materials and Google Patents, covering 2001–2026. Anatomical features, materials, active pharmaceutical ingredients, release profiles, and adverse events were analyzed. **Results:** Seventy-one sources were included. Occlusive plugs without an active pharmaceutical ingredient demonstrate premature expulsion in up to 57.4% of cases and bacterial colonization in 44%. Drug delivery systems provide release from 7 days (PEGDA hydrogels) to 3 months (Eximore, Ocular Therapeutix™). DEXTENZA^®^ (dexamethasone) is FDA-approved for postoperative inflammation, whereas pivotal trials of travoprost (OTX-TP) and latanoprost systems (L-PPDS, EXP-LP) did not demonstrate superiority over placebo or eye drops. In situ systems eliminate size-fitting requirements but face challenges related to gelation control and biodegradation. **Conclusions:** We propose the following candidate CQAs: retention (>80% over 4 weeks), swelling degree (30–60%), controlled burst release (<40% within 24 h), and mechanical compatibility. The proposed QTPP matrices for punctal, intracanalicular, and in situ systems may guide the development of ophthalmic drug delivery platforms.

## 1. Introduction

Over 2.2 billion people worldwide suffer from vision impairment or blindness, underscoring the global burden of ocular disease [1]. Eye drops remain the most widely used dosage form in ophthalmology and are first-line therapy for many diseases [2], and account for approximately 90% of commercially available ophthalmic preparations on the global market. Despite their widespread use in clinical practice, the effectiveness of topical drop therapy is limited by several anatomical and physiological features of the eye. The bioavailability of such therapy typically ranges from 0.5% to 5%. Due to the limited capacity of the precorneal space, only a small volume of the drug formulation can be applied to the ocular surface; moreover, a considerable proportion of the administered dose, approximately 60% of the active pharmaceutical ingredient (API), is eliminated through reflex blinking and nasolacrimal drainage [3]. An additional limitation is the presence of preservatives in some eye drops, which may have toxic effects on the tissues of the ocular surface [4].

Many ophthalmic diseases, including glaucoma, dry eye disease (DED), and chronic inflammatory ocular surface diseases, require long-term and often multiple daily drug administration [5]. Such treatment regimens reduce patient adherence and increase the likelihood of errors in eye drop use. According to published studies, 39.2% of patients forget to administer eye drops, 92.6% use them incorrectly, and only 20–30% maintain hygienic technique without the bottle tip touching the ocular surface [6,7]. These findings indicate that the clinical effectiveness of topical drop therapy is constrained not only by low bioavailability but also by patient-related behavioral factors, including incorrect administration technique and poor treatment adherence. Consequently, dosage forms with prolonged action and reduced dosing frequency are regarded as a promising strategy for improving adherence in patients with chronic and degenerative eye diseases [8].

Patient-centered study results also confirm significant inconvenience associated with long-term eye drop therapy. In a single-center anonymous survey by Weber et al. conducted among 102 glaucoma patients receiving topical hypotensive drugs, 35.3% reported that eye drops disrupted their lives, 41.2% expressed concern about their use, and 61.8% preferred alternative ophthalmic drug delivery systems over continued eye drop therapy. The most important characteristics in choosing alternative systems were efficacy, duration of effect, and minimal risk of adverse effects. At the same time, patients expressed concern about surgical interventions and a strong preference for non-invasive or minimally invasive approaches [9].

To overcome these limitations, alternative ophthalmic drug delivery systems are currently being developed, aimed at increasing therapeutic activity, prolonging drug contact time with ocular tissues, reducing the frequency of administration, and decreasing toxicity. Contemporary delivery systems include microemulsions, nanoparticle solutions, multicomponent carrier systems, ocular films, contact lenses, collagen shields, implants, and in situ-forming gels. Among minimally invasive approaches, systems placed in the lacrimal punctum or lacrimal canaliculus are of particular interest, as they exploit the anatomical features of the lacrimal drainage system to achieve prolonged retention of the dosage form and targeted drug release. For occlusive devices, an additional benefit is the reduction in tear drainage through the nasolacrimal duct, which promotes increased tear film residence time on the ocular surface and may be especially significant in the treatment of ocular surface diseases [10].

In a questionnaire study by Chan et al. including 250 Chinese patients from Singapore with glaucoma, the acceptability of three routes of sustained delivery of antiglaucoma drugs was assessed: subconjunctival, intracameral, and punctal. The punctal route of administration was accepted by many respondents—63.2% of patients, and 48.0% selected punctal plug placement as the most preferred option among the proposed delivery systems [11].

Thus, the development of minimally invasive drug delivery systems intended for placement in the lacrimal drainage system represents a promising direction for replacing conventional topical drop therapy.

However, the available literature lacks a systematic analysis of critical quality attributes (CQAs) for devices placed in the lacrimal drainage system, performed from the perspective of quality by design (QbD) methodology. The present review addresses this gap: based on anatomical constraints, clinical data, and regulatory requirements, we formulate the target product quality profile (QTPP) for punctal plugs, intracanalicular inserts, and in situ-forming systems, and identify the minimum necessary CQAs, providing practical guidance for developers of ophthalmic drug delivery systems.

The objective of this review is to analyze the relevant scientific data on punctal and intracanalicular delivery systems and occlusive devices, to characterize their composition, and to substantiate critical quality attributes for development considering the anatomical and physiological features of the lacrimal drainage system.

## 2. Materials and Methods

This is a narrative review conducted to analyze and synthesize scientific, clinical, regulatory, and technological data on punctal and intracanalicular ophthalmic systems intended for placement in the lacrimal punctum, lacrimal canaliculus, or the lacrimal drainage system. The methodological approach was structured to identify, evaluate, and synthesize relevant literature in the following areas: anatomical and physiological features of the lacrimal drainage system; design and clinical characteristics of occlusive devices; composition, mechanism of action, release profile, efficacy, and safety of punctal and intracanalicular drug delivery systems; in situ-forming systems; and critical quality attributes relevant to the development of such systems.

### 2.1. Literature Search Strategy

The literature search was carried out using the electronic databases PubMed, Scopus, Web of Science, Google Scholar, and ClinicalTrials.gov, as well as open regulatory, patent, and informational sources, including FDA materials, Google Patents, clinical trial data, and official manufacturer information. The primary search covered publications from 2001 to 2026. The last literature search was performed on 11 June 2026. Additionally, earlier fundamental and clinically significant studies were considered if they contained data on lacrimal drainage anatomy, punctal occlusion, plug complications, or served as primary sources for describing specific technologies.

The following keywords and their combinations were used: “punctal plug”, “punctal occlusion”, “intracanalicular insert”, “intracanalicular drug delivery”, “lacrimal canaliculus”, “lacrimal drainage system”, “ocular drug delivery”, “ophthalmic drug delivery”, “sustained release”, “controlled release”, “drug-eluting punctal plug”, “hydrogel”, “bioresorbable hydrogel”, “in situ gel”, “in situ-forming hydrogel”, “dry eye disease”, “glaucoma”, “dexamethasone intracanalicular insert”, “DEXTENZA”, “travoprost punctum plug”, “cyclosporine intracanalicular insert”, “latanoprost punctal plug”, “canaliculitis”, “dacryocystitis”. Boolean operators AND/OR were applied to expand the search.

### 2.2. Inclusion and Exclusion Criteria

Full-text scientific publications in Russian and English, clinical studies, preclinical and in vitro studies, patent documents, registration materials, and official manufacturer data were included if they contained information on the composition, design, mechanism of action, release duration, retention, efficacy, safety, or complications of punctal and intracanalicular systems.

The inclusion criteria were the presence of data on systems intended for insertion or fixation in the lacrimal punctum or lacrimal canaliculus; description of material, geometry, occlusion or drug delivery mechanism; presence of experimental, clinical, regulatory, or patent data; relevance to the objectives of this review.

Exclusion criteria were: publications not related to punctal or intracanalicular delivery systems; studies on non-ophthalmic or irrelevant delivery systems; publications lacking data on composition, design, mechanism of action, efficacy, safety, or system characteristics; review articles, letters, and editorials without primary data; duplicate publications or materials with overlapping data; sources for which full text or sufficient open information for analysis was unavailable.

### 2.3. Source Selection and Systematization

**Figure 1:**
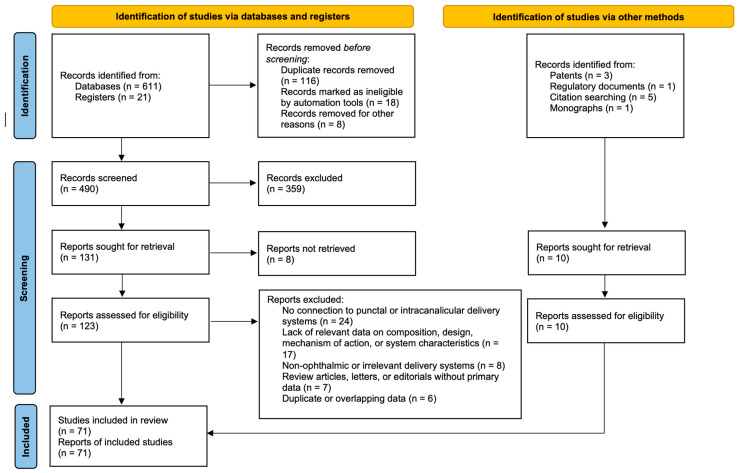
PRISMA flow diagram.

This review was not registered in PROSPERO or any other systematic review registry, as it was conducted as a narrative review and did not meet the criteria for prospective registration. A separate review protocol was not prepared. To improve the transparency of literature selection and reporting, a flow diagram based on the PRISMA 2020 statement was prepared (Figure 1), reflecting the main stages of identification, screening, full-text source evaluation, and inclusion in the review. A total of 642 records were identified from databases, registers, and additional sources: PubMed—249, Scopus—156, Web of Science—98, Google Scholar—93 (total databases—611), ClinicalTrials.gov—21, and additional sources, including patents (*n* = 3), regulatory documents (*n* = 1), citation searching (*n* = 5), and monographs (*n* = 1)—10.

Before the screening stage, 142 records were excluded: duplicate records—116, records marked as ineligible using predefined automated filtering criteria—18, and records removed for other reasons—8. Subsequently, 490 records were evaluated by title and abstract, of which 359 were excluded: 258 records were excluded using predefined automated pre-screening criteria, and 101 records were excluded after manual screening. Abstract screening and full-text eligibility assessment were performed independently by two authors (K.S.L. and N.O.B.); disagreements regarding source inclusion were resolved through discussion with the senior author (E.O.B.).

Full-text versions were sought for 131 publications and documents. Full texts of 8 sources were not obtained due to unavailability. Thus, 123 publications and documents were evaluated for compliance with the inclusion criteria by full text or available documentary data. All 10 sources identified via other methods were successfully retrieved and assessed for eligibility.

After full-text assessment, 62 sources identified via databases and registers were excluded for the following reasons: no connection to punctal or intracanalicular delivery systems—24; lack of relevant data on composition, design, mechanism of action, or system characteristics—17; studies of non-ophthalmic or irrelevant delivery systems—8; review articles, letters, or editorials without primary data—7; and duplicate or overlapping data—6.

The final qualitative synthesis included 71 studies and documents. These sources were used to analyze the anatomical and physiological rationale for development, device design features, materials, mechanisms of action, drug release characteristics, efficacy, safety, and critical quality attributes of punctal and intracanalicular ophthalmic systems.

### 2.4. Data Extraction and Synthesis

Primary source selection was conducted by title, abstract, and keywords. In the next stage, full texts of publications and Supplementary Materials were analyzed, including clinical trial data, patents, prescribing information for approved drugs, and manufacturer data. Sources were grouped by thematic sections of the review: anatomy and physiology of the lacrimal drainage system; occlusive devices without active pharmaceutical ingredient; punctal drug delivery systems; biodegradable intracanalicular systems; in situ-forming systems; target product quality profile and critical development parameters.

Data extraction was performed by the authors using predefined categories relevant to the objectives of the review. Extracted information was checked during data synthesis and discussed within the author group.

For included sources, the following data were extracted: system type, device location, material or polymer matrix, active substance, retention mechanism, occlusion or phase transition mechanism, predicted or established release duration, development stage, study design, primary efficacy endpoints, retention rate, safety profile, complications, and limitations affecting further technology development.

Data were synthesized qualitatively. Given the heterogeneity of the included sources, differences in study designs, and the simultaneous use of clinical, experimental, patent, regulatory, and manufacturing data, a meta-analysis was not performed.

### 2.5. Evidence Level Assessment

Given the narrative nature of the review and the high heterogeneity of the included sources, a formal risk-of-bias or certainty-of-evidence assessment using tools such as GRADE or the Cochrane Risk of Bias tool was not performed. However, the study design, sample size, presence of a control group, stage of clinical development, completeness of composition and methodological descriptions, and type of data source were considered when interpreting the evidence.

The highest priority in synthesizing the results was given to randomized controlled clinical trials, prospective clinical studies, registration materials, and full-text original research. Patent documents and manufacturer materials were used primarily to describe the composition, design, mechanism of action, and technological features of systems, particularly when clinical trial results had not been published in peer-reviewed literature.

### 2.6. Data Synthesis and Substantiation of Critical Quality Attributes

For each group of systems, common technological and biopharmaceutical patterns were analyzed, including the influence of device geometry, mechanical properties, swelling degree, biodegradation, mucoadhesion, release profile, and local tolerability on efficacy and safety. Special attention was paid to parameters that may be considered critical for the development of punctal and intracanalicular systems: device size and shape, compliance with the anatomy of the lacrimal punctum and canaliculus, retention, occlusion control, possibility of visual monitoring or removal, material biocompatibility, resistance to bacterial colonization, reproducibility of drug release, system stability, and absence of significant irritant effects.

Based on comparison of clinical, preclinical, in vitro, patent, and regulatory data, parameters significant for forming the target product quality profile of punctal and intracanalicular ophthalmic delivery systems were identified.

### 2.7. Use of Artificial Intelligence Tools for Figure Preparation

**Figure 2:**
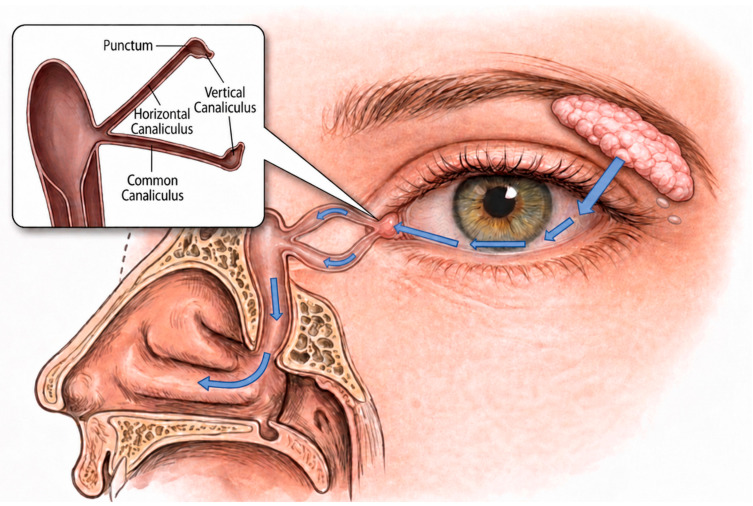
Structure of the drainage system.

**Figure 3:**
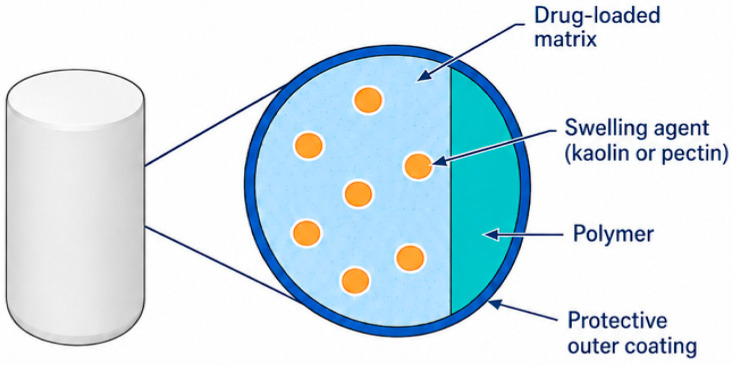
Structure of the EXP-LP latanoprost delivery device.

Generative artificial intelligence tools—specifically Claude Opus 4.6 (Anthropic) and ChatGPT GPT-5.4 (OpenAI)—were used only to assist in generating and iteratively refining preliminary graphical drafts of the anatomical and device schematics presented in Figure 2 and Figure 3 (the lacrimal drainage system and the EXP-LP latanoprost delivery device, respectively). The AI-assisted graphical drafts were subsequently reviewed, manually edited, and finalized by the authors in Microsoft PowerPoint to ensure anatomical and technical accuracy. No generative AI tools were used to produce the scientific content, data analysis, conclusions, or references of the manuscript. The authors take full responsibility for the accuracy, scientific validity, and integrity of all graphical content included in this review.

## 3. Anatomy and Physiology of the Lacrimal Drainage System

The lacrimal apparatus can be anatomically and functionally divided into three sections: the tear-producing section (lacrimal gland and accessory lacrimal glands), the tear-receiving section (conjunctival sac with the lacrimal rivus, lacrimal caruncle, plica semilunaris, and lacrimal lake), and the lacrimal drainage section, which includes the lacrimal puncta, lacrimal canaliculi, lacrimal sac, and nasolacrimal duct [12].

Tear fluid produced by the lacrimal glands spreads over the surface of the cornea and conjunctiva. When the eyelids close, the upper and lower lacrimal puncta contact the lacrimal lake, promoting entry of tear fluid into the drainage system, with approximately 90% of the fluid passing through the lower lacrimal canaliculus [13], and then entering the lacrimal sac and draining through the nasolacrimal duct (Figure 2).

The drainage system begins with the upper and lower lacrimal puncta—small orifices 0.2–0.3 mm in diameter, usually oval, visible only when the eyelid is retracted [12,13]. Behind the puncta are the upper and lower lacrimal canaliculi, each comprising a short vertical segment followed by a horizontal segment. According to anatomical studies, the length of the vertical segment is approximately 2.4 ± 0.5 mm, and its width is 1.6 ± 0.5 mm, while the mean length of the horizontal segment reaches 9.2 ± 2.3 mm with a width of approximately 0.5–0.6 mm [13,14]. The diameter of the lacrimal punctum and the lumen of the lacrimal canaliculus are of primary importance for the design of punctal and intracanalicular systems. Furthermore, blinking, tear drainage, and eyelid movement create mechanical loads that affect fixation, patient comfort, and the risk of migration or premature expulsion of the system. These parameters must be considered by developers of medical devices and drug delivery systems when justifying the target product quality profile (QTPP) and critical quality attributes (CQAs).

The lacrimal drainage system ensures tear drainage, regulates tear dynamics, and participates in maintaining tear film homeostasis [15]. Regulating tear outflow through occlusive devices increases the residence time of tear fluid and drugs on the ocular surface. This principle is used both in the treatment of ocular surface diseases and in the development of sustained local drug delivery systems.

## 4. Occlusive Devices (Without API)

Occlusive devices are small medical devices intended for partial or complete restriction of tear drainage through the lacrimal drainage system [16]. They are manufactured from biocompatible materials and inserted into the lacrimal punctum, as punctal plugs, or into the lacrimal canaliculus, as intracanalicular plugs. Such devices can provide temporary or long-term occlusion, the duration of which varies from several days to several months.

Occlusion represents a non-pharmacological treatment approach for dry eye syndrome, indicated when artificial tears fail to provide sufficient relief. The primary principle of the method is to reduce tear drainage, increase tear film residence time on the ocular surface, and improve tear film stability [17]. The clinical significance of punctal occlusion has also been confirmed in severe keratoconjunctivitis sicca associated with Sjögren syndrome: Mansour et al. revealed that lacrimal punctum occlusion may contribute to improvement of the ocular surface in patients with severe aqueous-deficient dry eye [18].

Additionally, this method can be used to improve the retention of topical medications on the ocular surface [19]. By reducing tear drainage, the contact time of eye drops with the ocular surface is prolonged, which may contribute to improved efficacy of topical therapy. Thus, occlusive devices can be viewed not only as devices that reduce tear drainage but also as a technological platform for the development of local drug delivery systems.

### 4.1. Punctal Plugs

Punctal plugs are inserted into the lacrimal punctum, allowing visual monitoring of their position and, if necessary, easy removal [20]. The design of such devices typically includes the following common elements: a cap (semi-permeable or impermeable membrane with one or more pores), a body (impermeable to drugs and tear fluid), and a nose (facilitating device insertion) [10].

By duration of residence in the lacrimal punctum, punctal plugs may be classified as temporary or permanent [10]. Temporary plugs are used for short-term occlusion, assessment of procedure tolerability, or temporary reduction in tear drainage. Permanent plugs are intended for long-term restriction of tear drainage, reduction in dry eye symptoms, and prolongation of tear fluid and topical medication residence time on the ocular surface.

Many permanent punctal plugs are commercially available, differing in size, shape, surface texture, shaft design, cap size, and insertion mechanism. Most devices are made of silicone, reflecting its biocompatibility, elasticity, and durability during long-term placement in the lacrimal drainage system.

Table 1 presents the main models of permanent punctal plugs used in clinical practice, their design features, and data from studies evaluating the efficacy and safety of these devices, including possible complications arising from long-term use.

Analysis of permanent punctal plug designs demonstrates that one of the key requirements for plug selection and development is reliable retention in the lacrimal punctum. Insufficient fixation increases the risk of premature extrusion, migration into the lacrimal canaliculus, and may cause damage to the drainage system, thereby contributing to canaliculitis, dacryocystitis, and pyogenic granuloma—a reactive granulation tissue overgrowth likely associated with chronic tissue irritation around the occluder [28]. This condition may be accompanied by an inflammatory reaction and generally requires plug removal to eliminate the source of irritation.

Design parameters of the plug, including body shape, presence of ribs or expansions, contact area with tissues, material rigidity, surface smoothness, and cap size, directly affect retention, patient comfort, and risk of complications. Conical shape, ribbed shaft, and materials of moderate rigidity may contribute to reducing the likelihood of early extrusion. However, excessive rigidity, irregular surface, or device size mismatch with the anatomical parameters of the lacrimal punctum may increase the risk of mechanical irritation, inflammatory reaction, and premature plug loss.

Equally important is proper sizing of the punctal plug. A mismatch between device diameter and punctum size may lead to either insufficient fixation and extrusion or excessive pressure on surrounding tissues. Manufacturers therefore offer extended size ranges for punctal plugs, and personalized device design—including 3D printing to individual lacrimal drainage system parameters—represents a promising direction for further development [29].

Since punctal plugs are minimally invasive medical devices, ease of insertion and removal, and reduction in the foreign body sensation experienced by the patient are also important requirements. These characteristics can be ensured by a smooth cone surface, designs that expand after insertion into the lacrimal punctum, and an adapted cap shape that follows eyelid anatomy.

### 4.2. Intracanalicular Plugs

Intracanalicular plugs are positioned inside the lacrimal canaliculus, distinguishing them from punctal plugs fixed in the lacrimal punctum. This location makes the device less visible and potentially more comfortable for the patient, but complicates visual monitoring of position and may complicate removal when necessary. Their design features, materials, and clinical characteristics are presented in Table 2.

The main requirements for intracanalicular plug development arise from the frequent complications also described in the table above. The material must be biocompatible, non-allergenic, and simultaneously resistant to bacterial colonization, as this may lead to canaliculitis and dacryocystitis. It is important that the plug does not cause a pronounced foreign body effect or traumatize canalicular tissues. The design must provide partial rather than complete occlusion of the lacrimal pathway to prevent tear stasis and consequent infection. The surface should be smooth to minimize irritation and granulation tissue formation. Equally important are position monitoring and the ability to remove the plug without trauma. It must be reliably fixed to minimize the risk of device migration, which requires surgical extraction.

Thus, an intracanalicular plug must combine stable retention or controlled swelling, partial or managed occlusion, resistance to bacterial colonization, and the possibility of safe removal.

## 5. Punctal Drug Delivery Systems

Punctal plugs can be used not only as occlusive devices but also as drug-loaded delivery systems providing sustained drug release over several weeks or months [36]. This ensures unidirectional API delivery and prolongs its contact with the ocular surface. Drug solutions, suspensions, emulsions, nanoparticles, micro- and nanoparticles, or liposomal suspensions may potentially be loaded into the body of a punctal plug [10]. Several such designs have been described in the literature, along with clinical trials evaluating their efficacy.

### 5.1. Evolute^®^ Punctal Plug Delivery System (Mati Therapeutics, Austin, Texas, USA)

The Evolute^®^ punctal plug delivery system, developed by Mati Therapeutics (Austin, TX, USA), is a silicone non-invasive system sealed on the sides to prevent drug washout into the nasolacrimal duct. The platform was considered for application in several therapeutic areas, including glaucoma, ocular hypertension, postoperative pain and inflammation, allergic diseases, and dry eye syndrome. The design represents a cosmetically inconspicuous device, colored for easy physician identification, with an L-shaped form facilitating placement and improving retention. According to the developer, the platform could be adapted for delivery of various active substances.

Latanoprost punctal plug delivery system (L-PPDS) is one of the products developed by Mati Therapeutics for glaucoma treatment. The plug body was filled with a latanoprost polymer matrix surrounded by non-biodegradable silicone [37]. The delivery system was designed to provide sustained API release over 30 days.

Patent US10632012B2 describes the composition and characteristics of L-PPDS in detail: length from 4 mm to 8 mm, diameter from 0.7 mm to 0.9 mm, with an angle of approximately 90 degrees between the vertical and horizontal segments. The cavity for the therapeutic agent is in the vertical segment (head) of the punctal plug, with a diameter of 0.2 mm to 1.4 mm.

The device body is made of silicone. The material must be soft enough to ensure patient comfort while sufficiently elastic for retention in the lacrimal drainage system. The API cavity contains latanoprost as the active substance; a special medical-grade silicone that slowly releases the API may be used as a matrix, with phosphatidylcholine as an excipient for formulation stabilization [38].

Most L-PPDS clinical trial results remain unpublished. Despite this, the scientific literature describes a Phase I study involving five patients that established safety, good tolerability, and a reduction in intraocular pressure of approximately 30%. Phase II clinical trial results for the delivery system loaded with 44 µg and 81 µg of latanoprost (L-PPDS) showed a mean intraocular pressure (IOP) reduction with the 44 µg dose of 3.5 mmHg at the end of 4 weeks, with 36% of patients achieving an IOP reduction of more than 5 mmHg. 78% of patients completed the 4-week follow-up and retained L-PPDS in the lacrimal punctum. Adverse effects included pruritus, ocular irritation, increased lacrimation, and ocular discomfort [39]. Comparison of L-PPDS with eye drops showed insufficient efficacy of the latanoprost punctal plug [40].

Nepafenac punctal plug delivery system (N-PPDS) is another punctal plug delivery system containing nepafenac, a non-steroidal anti-inflammatory drug, from Mati Therapeutics. In a multicenter, randomized, parallel-arm, placebo-controlled Phase II clinical trial (NCT03496467), the safety and efficacy of N-PPDS were evaluated compared with a placebo punctal plug delivery system (p-PPDS) for the control of postoperative ocular pain and inflammation after cataract surgery. The study enrolled 56 patients, of whom 38 were in the N-PPDS group (experimental group) and 18 in the p-PPDS group (control group). At three days, the proportion of pain-free patients was 69% (22 of 32) in the experimental group and 38% (6 of 16) in the control group. At 7 days, the trend was maintained: absence of pain was noted in 67% (24 of 36) of N-PPDS patients and 31% (5 of 16) in the placebo group. The plug retention rate was 98% (55 of 56) at 14 days [41].

At present, clinical trials of the Evolute^®^ platform have stalled at Phase II. There are no publicly available data on Phase III trials, new peer-reviewed publications, or FDA registration for the described systems. The devices showed generally favorable retention in the lacrimal drainage system due to the L-shaped design and appropriate material rigidity. However, N-PPDS showed no significant advantage over placebo, and L-PPDS was inferior to topical eye drops in IOP-lowering efficacy. The reasons for L-PPDS underperformance have not been formally established. One possible explanation may relate to the limited drug reservoir capacity of punctal systems, which can restrict total API loading; however, inadequate release kinetics, insufficient ocular surface bioavailability, dose–response limitations, and disease-specific pharmacodynamic requirements may also have contributed. Therefore, drug loading should be considered a plausible contributing factor, but not a proven root cause of the limited clinical efficacy of L-PPDS.

### 5.2. Eximore Pack and Release Ophthalmic Drug Delivery Platform

Eximore is a biopharmaceutical company specializing in the development of innovative therapies. One of its technologies was a patented multilayer ‘pack & release’ platform, which is a non-degradable punctal plug loaded with API.

EXP-LP is a device placed in the lacrimal punctum to provide sustained release of latanoprost. According to patent US20210137942A1, published in 2021, the device comprises a complex composite structure containing several elements: inert porous materials with high surface area and low density, such as fumed silica, silica gel, activated charcoal, and zeolite, onto which latanoprost is adsorbed. A swelling agent—kaolin or pectin—may also be included in the matrix. A binding element represented by epoxy resin or polyurethane is used to form a rigid structure. Furthermore, the entire device surface or part of it may be coated with a protective layer (e.g., parylene or butyral) (Figure 3). The coating may be either continuous or perforated with microholes of 0.5–5 microns. The patent describes two compositions: the first, based on an epoxy matrix with 36% latanoprost content, and the second, a dispersed composite polyurethane matrix containing 14% API. The possibility of creating a combined system with latanoprost and timolol is also described.

During development, the latanoprost release profile from samples into phosphate-buffered saline during incubation at 37 °C over various time periods, as well as daily API release over three months, was determined. The API release from punctal plugs with different coating types was also compared [42].

According to company data from Eximore, in vitro preclinical studies showed that EXP-LP can provide controlled API release over three months. In animal studies, the device demonstrated a favorable safety profile and a 50% reduction in IOP during the first eight weeks, with no serious adverse effects.

Furthermore, in a prospective, single-center, open-label, controlled, non-randomized clinical study, the safety, plug retention, and preliminary efficacy of EXP-LP were evaluated. The device was tested in two sizes and doses: a ‘large plug’ (diameter 1.08 mm, API content 450 µg) and a ‘small plug’ (diameter 1.06 mm, API content 250 µg). The study included 40 patients. The latanoprost punctal plug was inserted into one eye, while Xalatan^®^ 1 drop once daily was instilled into the other eye. This study was completed in 2019, but its results were not published [43].

EXP-TC is another ‘pack & release’ system representing a punctal plug with a dual function: mechanical occlusion of tear drainage and targeted drug therapy for dry eye syndrome. The device is molded from a medical-grade composite containing tacrolimus, an immunosuppressive macrolide drug. The EXP-TC design includes a flattened head that remains visible at the lacrimal punctum and a central shaft tapering toward the nose. Drug release is activated upon interaction of tear fluid with the plug. This mechanism is designed to ensure continuous API delivery over three months.

According to company data from Eximore, in vitro studies demonstrated the plug’s ability to provide controlled drug release. Over 60 days, the amount of API released exceeded the minimum therapeutic threshold of 0.6 µg/day. In animal studies, the device demonstrated a favorable safety profile and better outcomes than macrolide eye drops, with no serious adverse effects (edema or opacities).

To evaluate safety, tolerability, device retention, and preliminary efficacy, a prospective, single-center, open-label, non-randomized Phase I study was conducted in 2023 involving 20 patients with moderate and severe dry eye disease. Preservative-free 0.15% sodium hyaluronate solution (Hyabak^®^) was used as an active control. The results of this study were not published [44].

### 5.3. PEGDA-Based 3D-Printed Hydrogel Punctal Plugs

Xu et al. [45] developed various formulations of drug-loaded punctal plugs based on polyethylene glycol diacrylate (PEGDA) hydrogels using digital light processing (DLP) 3D printing. Photopolymer solutions based on polyethylene glycol diacrylate (PEGDA) were prepared to form matrices. Dexamethasone, PEGDA, and polyethylene glycol 400 (PEG 400) were incorporated into the compositions. The study was conducted with four main formulations: D10 and D20, where PEGDA was the sole polymer component (with different drug loads), and D10PEG and D20PEG, in which PEG 400 was added to PEGDA in a 4:1 (*w*/*w*) ratio. PEG 400 was used to create a semi-interpenetrating network and increase matrix hydrophilicity, which could facilitate drug diffusion from the polymer matrix.

To evaluate system efficacy and safety, plug surface morphology was examined by scanning electron microscopy (SEM), cytocompatibility was assessed using the BALB/3T3 fibroblast cell line, API release rate in a dynamic flow in vitro model simulating anterior ocular segment conditions was studied, and dexamethasone content was determined by high-performance liquid chromatography (HPLC) after extraction from sectioned plugs in ethanol. The tests demonstrated that increasing dexamethasone concentration from 10% to 20% was accompanied by a slight increase in surface roughness; D10PEG exhibited pronounced burst release on day 1 with complete drug release within 4 days, while D20PEG provided more prolonged release over a week. The slower release rate of D20PEG was explained by the poor aqueous solubility of dexamethasone, which served as the limiting factor. Cytocompatibility depended on composition: control plugs maintained 100% cell viability, while D10PEG and D20PEG reduced it by approximately 50% at 24 h, attributed to the cytotoxicity of free dexamethasone at high concentrations and the possible presence of unreacted monomers, indicating the need for additional washing steps to improve monomer conversion and biocompatibility of the delivery systems.

Table 3 summarizes the main API-loaded punctal plug delivery systems discussed below.

## 6. Intracanalicular Hydrogel Drug Delivery Systems

In addition to punctal plugs, intracanalicular hydrogel systems represent a promising platform for local sustained drug delivery. The crosslinked polymer structure of hydrogels allows drugs to be retained in the matrix and released gradually [46], while biocompatibility, high water absorption capacity, and adjustable mechanical properties make such materials attractive for ophthalmic delivery systems [47]. In the context of intracanalicular application, the key property of hydrogels is their swelling capacity: upon contact with physiological fluids, the hydrogel absorbs water, expands, and can adapt to the shape of the canaliculus, promoting device fixation and reducing dependence on pre-sizing. An additional advantage of biodegradable polymer platforms is the possibility of gradual degradation after the depot function is fulfilled, potentially reducing the need for subsequent system removal and repeated procedures [48].

Hydrogel plugs can be used either in dried form or directly as a solution for delivering drugs of various classes, including steroids, NSAIDs, antibiotics, and pressure-lowering agents [49].

### 6.1. ELUTYX™ (Ocular Therapeutix™)

ELUTYX™—a patented hydrogel technology based on polyethylene glycol (PEG) developed by Ocular Therapeutix™—is a biodegradable hydrogel platform for local drug delivery through the lacrimal canaliculus. Small molecules previously FDA-approved can be incorporated into the hydrogel matrix. Biodegradation of these systems is governed solely by tear fluid temperature and pH. These parameters are sufficiently stable, so the hydrogel dissolution time can be predicted. According to the developer, the platform’s advantages include biodegradation without generating an acidic environment, soft consistency, easy removal if necessary, and a reduced risk of impurities, allergens, or immunogenic components owing to the synthetic nature of the materials.

Ocular Therapeutix™ has several products based on its patented technology. OTX-TP is one of them and represents an intracanalicular plug for travoprost delivery to the ocular surface over three months [50]. The device is based on a polyethylene glycol hydrogel rod in which travoprost particles encapsulated in polylactic acid microspheres are embedded. Insertion can be performed into the upper or lower lacrimal canaliculus, where fluid absorption and swelling occur. The rod is impregnated with fluorescein to facilitate visual monitoring.

Clinical trials of the OTX-TP demonstrated its potential efficacy and safety in early development stages. In an initial prospective study involving 17 patients (26 eyes) with primary open-angle glaucoma or ocular hypertension, plug retention at 10 days post-implantation was 100%, and IOP reduction reached 6.2 mmHg (23%) at 8:00 a.m., 5.4 mmHg (21%) at 10:00 a.m., and 7.5 mmHg (28%) at 16:00. By day 30, plug retention had declined to 42% and the IOP-lowering effect decreased to approximately 16% [51].

In a randomized double-masked Phase II study involving 73 patients, OTX-TP in combination with artificial tears was compared with timolol 0.5% combined with a placebo punctal plug. Both groups showed clinically significant IOP reduction, but the effect was more pronounced in the timolol group: 6.4–7.6 mmHg compared with 4.5–5.7 mmHg in the OTX-TP group. The greater IOP reduction in the timolol group is presumed to be related to prolonged drug contact time with the ocular surface due to the presence of the plug. OTX-TP plug retention was 91% on day 60, 88% on day 75, and declined to 48% by day 90 [52]. The most common adverse effects with this system were foreign body sensation (38.5%), pruritus (15.4%), and epiphora (3.8%) [53].

Subsequently, a large placebo-controlled Phase III study was conducted involving 554 patients at more than 50 centers. However, it did not demonstrate statistically significant superiority of OTX-TP over placebo in IOP reduction at 2, 6, and 12 weeks of follow-up. Among adverse effects, dacryocanaliculitis was noted in 7% of patients in the OTX-TP group compared with 3% in the placebo group. Due to the absence of a significant efficacy advantage, further development of the OTX-TP system was discontinued [51,52].

Another ELUTYX™ technology-based development was OTX-CSI—an intracanalicular insert containing cyclosporine as an immunomodulatory drug. The agent was intended for patients with moderate and severe dry eye syndrome. Cyclosporine release to the ocular surface occurred over approximately 3–4 months.

The efficacy, tolerability, and safety of OTX-CSI were evaluated in a randomized, multicenter, double-masked controlled Phase 1/2 clinical study. 153 patients were allocated into three subgroups: two receiving OTX-CSI 0.36 mg with different intended release durations (2–3 months and up to 4 months, respectively) and one receiving a placebo hydrogel insert. Patients were observed for 16 weeks. The primary endpoint was the Schirmer test at week 12, showing the increase in tear production in patients. OTX-CSI results showed no significant difference compared with the placebo group. Placebo group patients showed an increase of 2.24 to 3.08 mm, while patients receiving the cyclosporine intracanalicular insert showed an increase of 1.91 to 1.98 mm. Regarding safety, the OTX-CSI implant was generally well tolerated [54]. There is currently no information on further clinical trials.

OTX-DED is also an intracanalicular ophthalmic insert intended for the treatment of signs and symptoms of dry eye syndrome, but it contains dexamethasone as an API. The glucocorticosteroid release is provided over 2–3 weeks.

In a randomized double-masked controlled Phase II study, 166 patients were distributed into three groups. The first group received OTX-DED with 0.2 mg dexamethasone content (55 subjects), the second group received OTX-DED with 0.3 mg dexamethasone (56 subjects), and the third received a placebo insert (55 subjects). The primary efficacy endpoint was the assessment of bulbar conjunctival hyperemia change at day 15 from baseline. The mean change was −0.51 for the OTX-DED 0.2 mg group and −0.43 for the OTX-DED 0.3 mg group, compared with −0.21 in the placebo group [55]. Although the study was not initially powered to demonstrate statistical significance, the results showed statistically significant improvement (reduction in hyperemia) in the OTX-DED groups compared with the placebo group.

DEXTENZA^®^ is a commercially available, FDA-approved biodegradable intracanalicular insert and represents a 3 mm cylindrical-shaped, resorbable, sterile ophthalmic insert intended for insertion into the lower lacrimal canaliculus [56,57]. This insert has a fluorescent yellow color and contains 0.4 mg dexamethasone in a polyethylene glycol (PEG)-based hydrogel conjugated with fluorescein, with no antimicrobial preservatives. Excipients include 4-arm PEG, N-hydroxysuccinimidyl glutarate (20K), trilysine acetate, N-hydroxysuccinimide-fluorescein, dibasic sodium phosphate, monobasic sodium phosphate, and water for injection [56].

A single intracanalicular insert releases dexamethasone over 30 days after insertion and is intended for single use only. As this system is resorbable, it does not require removal; however, it can be extracted by saline irrigation or manual expression if necessary [58].

DEXTENZA^®^ demonstrated clinically significant efficacy in controlling postoperative pain and inflammation after cataract surgery. In two Phase III studies summarized by Walters et al., the absence of pain on day 8 was noted in 80.4% and 77.5% of patients in the dexamethasone-loaded intracanalicular insert groups versus 43.4% and 58.8% in the placebo groups, respectively. Statistically significant differences were also observed in anterior chamber inflammation reduction and a lower need for rescue therapy [59]. In a subsequent multicenter randomized double-masked Phase III study involving 438 patients, DEXTENZA^®^ also achieved its therapeutic endpoints: absence of anterior chamber cells on day 14 was observed in 52.3% of patients versus 31.1% in the placebo group, and absence of pain on day 8 in 79.6% versus 61.3%. The adverse event profile was comparable to placebo [60].

In four randomized placebo-controlled studies involving 567 patients with ocular pain and inflammation after ophthalmic surgery with a mean age of 68 years, adverse effects including anterior chamber inflammation (10%), elevated intraocular pressure (6%), reduced visual acuity (2%), as well as cystoid macular edema, corneal edema, eye pain, and conjunctival hyperemia (1% each) were identified. In a safety study for the treatment of itching in allergic conjunctivitis (*n* = 154, mean age 41 years), the most common reactions were elevated intraocular pressure (3%), increased lacrimation, eye discharge, and reduced visual acuity (1% each) [7]. Despite this, in real-world clinical practice, the intracanalicular dexamethasone insert also demonstrated high patient acceptability: 93% of patients were satisfied or very satisfied with the treatment, and 93% would prefer this system over postoperative eye drops [61].

### 6.2. Photo-Crosslinked Hydrogel Drug Delivery System (Lin, Wang et al.)

Lin et al. [62] developed a drug delivery system for the lacrimal duct based on a photocrosslinkable hydrogel consisting of four-arm polyethylene glycol norbornene (4aPEGNB) and methacrylate-modified hyaluronic acid (HAMA). Introduction of the methacrylate group into the hyaluronic acid chain allowed subsequent photocrosslinking under ultraviolet (UV) irradiation, yielding a higher-strength hydrogel [63].

In the delivery system with encapsulated cyclosporine A (CsA), the drug concentration was 10% and 30%, and a hydrogel with 10% tacrolimus was prepared for the FK506 system. To obtain plugs of the required shape, the hydrogel with encapsulated drug was introduced into silicone tubes with a defined inner diameter. After complete gelation under light irradiation (395 nm), the hydrogels in tubes were placed in an oven at 80 °C until fully dried.

Mechanical properties of hydrogels with API loading from 2 to 30% CsA and 10% FK506 were studied, as intracanalicular plugs are subjected to compressive loads in the lacrimal duct. The hydrogel revealed high strength at relatively low solid content (5%), but adding the drug reduced strength. Hydrogels with higher drug loading showed higher degradation rates and greater swelling degree: from 33.9% (without drug) to 49.5% (at 30% CsA). High drug concentration is presumed to interfere with the crosslinking reaction, increasing swelling, which may promote better device adaptation to the lacrimal canaliculus lumen.

In an in vitro API release study, the hydrogel plug showed an almost linear release curve over two months. By day 60, more than 30% of CsA and more than 80% of FK506 remained in the plug. The developers also concluded that increasing API content and reducing hydrogel density could enhance in vitro drug release.

In vivo drug release was studied in rabbits over 90 days. The study showed stable and sustained release attributed to low CsA solubility.

Therapeutic efficacy was demonstrated in two disease models. In the treatment of allergic conjunctivitis in rats, the use of tacrolimus plugs led to a rapid reduction in edema and hyperemia and a decrease in inflammatory cells. In dry eye syndrome in rabbits, cyclosporine plugs revealed a dual benefit: they not only mechanically retained tear fluid on the ocular surface but also actively suppressed inflammation, promoting goblet cell restoration and corneal healing.

The main biodegradable intracanalicular drug delivery systems, their composition, active substances, and key preclinical and clinical results are summarized in Table 4.

Analysis of clinically developed and experimental intracanalicular systems demonstrates that their efficacy is determined not only by drug selection but also by swelling degree, mechanical strength, degradation rate, canalicular retention, release profile, and local tolerability. The OTX-TP experience demonstrates that sustained release alone does not guarantee clinical benefit if the system does not ensure stable retention. Meanwhile, DEXTENZA^®^ confirms the possibility of successful regulatory implementation of a biodegradable intracanalicular insert when a reproducible release profile, acceptable safety, and clinically confirmed efficacy are present.

## 7. In Situ-Forming Intracanalicular Systems

In situ technology (from Latin ‘on site’) is currently one of the most widely discussed technologies for targeted drug delivery systems. Such systems may have various final forms—gels, implants, emulsions, films—but their unifying feature is a phase transition occurring locally at the application site in response to pathological or physiological stimuli. Thus, a drug administered in liquid form undergoes a phase transition at the application site to form a solid or semi-solid depot system with improved retention, resistance to natural clearance, and sustained drug release.

In situ gelling systems are particularly promising for ophthalmic delivery, as they are introduced as a solution and transition to their final form in the physiological conditions of the eye. This property facilitates uniform distribution of the polymer system over the ocular surface during blinking and prolongs drug contact time with ocular tissues [64].

In situ systems are most often classified by the nature of the stimulus promoting their formation into thermosensitive, ion-sensitive, pH-sensitive, photosensitive, moisture-activated, and phase-inversion types. Combinations of stimuli-sensitive polymers—known as multi-responsive systems—are often used for better system adaptation to in situ conditions [65].

### 7.1. In Situ-Forming Occluders

The property of stimulus sensitivity can be imparted to polymers that not only have suitable physicochemical properties but also provide sufficient pharmacological activity for the use of such systems in medicine. Thus, a subclass of in situ-forming intracanalicular systems can be distinguished that will not serve as drug carriers but can be successfully positioned as medical devices.

#### 7.1.1. Thermosensitive ‘Liquid’ Hydroxybutyl Chitosan Plug

Lin et al. [66] developed a ‘liquid plug’ strategy based on intracanalicular injection of hydroxybutyl chitosan (HBC) solution—a thermosensitive biomaterial capable of rapidly transitioning from liquid phase to hydrogel. In this development, this process is completed at body temperature (37 °C) in approximately 50 s.

The duration of therapeutic effect is related to the degradation rate of the absorbable material, as tear outflow through the drainage system increases as the material degrades. In testing HBC versus VisiPlug^®^, patients with dry eye disease may require relatively frequent HBC injection therapy to maintain efficacy. Based on clinical data, the effect is presumed to last at least 4 weeks, although more frequent administration may be required in some patients.

No significant differences in the rate or severity of adverse events were identified, apart from foreign body sensation (HBC: 0%; VisiPlug^®^: 9%). This is consistent with the developers’ suggestion that the HBC ‘liquid plug’ has several advantages over conventional plugs, primarily due to its individualized and adaptive nature. The method requires no size fitting and reduces the risk of complications such as spontaneous extrusion. Furthermore, HBC may have antibacterial activity, potentially reducing the risk of infection, and the material phase transition is accompanied by volume contraction, helping to avoid complete canalicular obstruction.

#### 7.1.2. In Situ-Forming Hydrogel Plug

Dai et al. [67] proposed an injectable in situ-forming SFMA/FTN hydrogel plug for temporary lacrimal pathway occlusion in dry eye syndrome. The formulation included silk fibroin methacrylate (SFMA) as a photocrosslinkable hydrogel matrix, fluorescent tracing nanoparticles FTN based on indocyanine green, and the photoinitiator lithium phenyl-2,4,6-trimethylbenzoylphosphinate (LAP), with the optimized formulation containing 8% SFMA, 0.02% FTN, and 0.4% LAP.

After intracanalicular injection of the liquid SFMA/FTN solution, the system was irradiated with blue light for 1 min, which initiated free-radical polymerization and led to the formation of a hydrogel plug conforming to the shape of the lacrimal canaliculus. The authors conducted physicochemical and morphological characterization of the system, including transmission electron microscopy (TEM), Fourier-transform infrared spectroscopy (FT-IR), ultraviolet–visible spectroscopy, SEM, assessment of gelation, swelling/degradation, and rheological properties. Gelation time decreased from 10.5 ± 1.0 to 3.3 ± 0.3 s as SFMA concentration increased from 5 to 10%, and hydrogels exhibited a porous structure, rapid photocuring, and in situ formation capability.

System efficacy was evaluated in vitro and in vivo: biocompatibility was confirmed using human corneal epithelial cells, hemolytic testing, and after subcutaneous administration in mice, and therapeutic action was evaluated in a rabbit dry eye model. After SFMA/FTN plug insertion, lissamine green dye did not pass from the eye to the nasal cavity, indicating complete occlusion of the lacrimal drainage system and cessation of tear drainage through the nasolacrimal pathway. This obstruction was temporary, as permeability partially recovered within 7 days, while the plug improved tear retention and reduced fluorescein corneal staining. In vivo imaging system (IVIS) fluorescence imaging demonstrated preservation of a localized signal in the lacrimal canaliculus region for up to 28 days, confirming the possibility of non-invasive monitoring of plug position and degradation.

### 7.2. In Situ-Forming Drug Delivery Systems

#### 7.2.1. Phase-Inversion System—Injectable CsA Organogel In Situ

Another example of an in situ system is the development by Ziqin Cao et al. of an injectable organogel aimed at creating an effective local delivery platform with sustained action. An organogel (or oleogel) is a semi-solid gel system where an organic liquid (oil or non-polar solvent) is retained within a three-dimensional gelator network [68]. The liquid phase is retained through hydrogen bonds, π-π stacking, or van der Waals forces.

Cyclosporine A (CsA) was used as API, and the gelling composition included stearic acid (a lipophilic gelator), injectable soybean oil (organic phase), and N-methyl-2-pyrrolidone (NMP) (co-solvent) in a ratio of 1.25:10:0.6 (*w*/*v*/*v*). This system provides phase transition from solution to gel after injection through diffusion of the anti-gelling co-solvent—such a system can be characterized according to the above classification as a phase-inversion in situ system.

The developed system rapidly gels in situ in the lacrimal canaliculi, causing their occlusion while simultaneously providing drug release, implementing a dual mechanism of action. In vitro experiments demonstrated a reduction in fluid flow to 52.78% at 2 min, while in vivo results showed complete lacrimal pathway occlusion and significant reduction in tear drainage.

Pharmacokinetic data demonstrated improved CsA bioavailability: Cmax values increased approximately 1.9-fold, and the area under the curve (AUC) increased 2.15–2.49-fold in ocular tissues compared with eye drops, confirming the efficacy of the developed system [69].

A consideration that merits attention for this system is the choice of N-methyl-2-pyrrolidone (NMP) as a co-solvent. NMP is a widely used, water-miscible pharmaceutical solvent that has been employed as a solubilizing vehicle in injectable depot formulations, which supports its applicability for in situ–forming systems. At the same time, NMP is a recognized local irritant and has been associated with reproductive toxicity at high systemic exposure, which is why its concentration and total administered amount require careful control. For a lacrimal-route in situ system, however, the administered volume and the resulting NMP load are very small and locally confined, so the principal concern is local tolerability of the canalicular tissue rather than systemic exposure. This favorable balance notwithstanding, the local safety and tolerability of NMP-containing organogels in the lacrimal drainage system have not yet been specifically established and should be confirmed in dedicated ocular safety studies.

#### 7.2.2. In Situ Lacrimal Duct Implant Based on a Biodegradable Thermoreversible Polymer Formulation

The investigated dosage form represents an in situ lacrimal duct implant with a biodegradable thermoreversible polymer formulation. The system is introduced into the lacrimal canaliculus as a solution, freely distributes throughout the lacrimal drainage system, and transitions from sol to gel upon temperature elevation in the inflammation zone to 38–40 °C.

When the temperature decreases to physiological values (approximately 37 °C), the polymer system undergoes a reverse gel-to-sol transition and is removed through the nasolacrimal duct. Such thermoreversibility reduces the risk of lacrimal canalicular and sac obstruction, as well as the likelihood of complications associated with impaired tear outflow.

Kolliphor^®^ P 407 poloxamer (BASF, Ludwigshafen am Rhein, Germany) was used as the base, with Kolliphor^®^ P 188 poloxamer, PEG 1500, hydroxyethylcellulose, hyaluronic acid, and the mucoadhesive Polycarbophil Noveon^®^ AA-1 used to form polyplexes. Nitrofuralum at 40 mg per 10 mL polymer base and diclofenac at 25 mg/mL were used as active components. Samples were prepared by the ‘cold’ method with subsequent storage in a refrigeration chamber for 7 days.

For in vitro assessment, a lacrimal duct and lacrimal sac model was created considering the anatomical features of the lacrimal drainage system at a 1:8 scale. The lacrimal canaliculus was modeled as a polymer tube 8 cm long and 0.4 cm in diameter; the lacrimal sac was made from polymer material. An artificial tear eye drop solution with hypromellose 0.5% and 4% type II porcine gastric mucin was used to simulate the mucous membrane and tear fluid. The model was thermostatted at 40 °C, after which the dosage form was administered by syringe (3–5 mL volume). Gelation time was recorded from the moment of sample introduction to the visual increase in dynamic viscosity. Additionally, pH, temperature, and gelation time were assessed, with the optimal pH value approaching tear fluid pH—7.4.

Criteria for selecting the optimal formulation were preservation of thermoreversible properties, gelation temperature for the inflammatory model in the range of 38.5–40 °C, minimum gelation time, and pH compliance with physiological values. The optimal formulation was recognized as containing poloxamer 407 18.0%, poloxamer 188 3.0%, and diclofenac 25.0 mg/mL. It was also noted that administration of the sample containing nitrofuralum may lead to lacrimal drainage system obstruction, tissue trauma, and exacerbation of pathological processes; therefore, this API was not included in the optimal formulation. The mean gelation temperature of this composition was 38.7 °C [70].

The main in situ-forming intracanalicular systems described above, including their composition, selection criteria, and key in vitro and in vivo results, are summarized in Table 5.

Thus, for in situ-forming intracanalicular systems, it is particularly important to control parameters determining their composition, mechanism and rate of phase transition, gelation conditions, pH, biocompatibility, biodegradation, degree of occlusion, API release profile, and duration of therapeutic effect. 

## 8. Target Product Quality Profile for Development of Products for Lacrimal Drainage System Placement

Modern development of pharmaceuticals and medical devices must be conducted in accordance with the strategy described in ICH Q8 (R2)—‘quality by design’—which describes approaches and tools for establishing conscious product quality from the preformulation or development stage.

The first stage should involve formation of the target product quality profile (QTPP)—a comprehensive list of product properties covering its clinical use, biological properties, physical and chemical characteristics, technological parameters, as well as consumer properties ensuring optimal biopharmaceutical parameters. Such a profile, given its broad scope, can only be developed by a multidisciplinary team—physicians, marketing specialists, technologists, drug developers, risk managers. For conventional dosage forms such as tablets, solutions, and capsules, QTPPs are well-described and rarely go beyond parameters describing route of administration, dose, and the complete set of pharmacopeial parameters. However, QTPP formation for non-pharmacopeial complex drug delivery systems (such as in situ systems) or medical devices may be a significantly limiting factor for implementing the QbD approach in their development.

In this section, based on the analyzed array of published data, we attempted to formulate typical target quality profiles for various products discussed above—punctal plugs (Table 6), intracanalicular plugs (Table 7), in situ occluders, and drug delivery systems (Table 8).

It is important to note that the described target/optimal values are often qualitative—due to the lack of representative published data. In such cases, the research team must independently establish quantitative metrics for optimal ranges of the described characteristics based on preliminary studies or literature review at the preformulation stage, and likely in consultation with clinical specialists.

Additionally, requirements related to ease of removal and monitoring of device position in the lacrimal drainage system may be imposed on medical devices. The system should be visible to the physician upon examination to assess its location in the lacrimal punctum or canaliculus, and should provide for the possibility of controlled and safe removal when necessary, without traumatizing surrounding tissues.

Following the QTPP elements presented in Table 6, Table 7 and Table 8, it is important to distinguish between the desired target profile of lacrimal drainage pathway systems and the development risks that may prevent this profile from being achieved. The proposed target values should therefore be interpreted as risk-informed development ranges rather than universally validated acceptance criteria. Their practical relevance depends on whether the system can maintain retention, provide reproducible drug release, remain biocompatible during residence in the lacrimal drainage pathway, and withstand manufacturing, sterilization, and clinical-use conditions. From a pharmaceutical development perspective, these requirements are closely linked to dosage-form design, material selection, swelling and degradation behavior, API loading and release kinetics, sterility assurance, and the translational gap between in vitro testing and in vivo performance.

**Figure 4:**
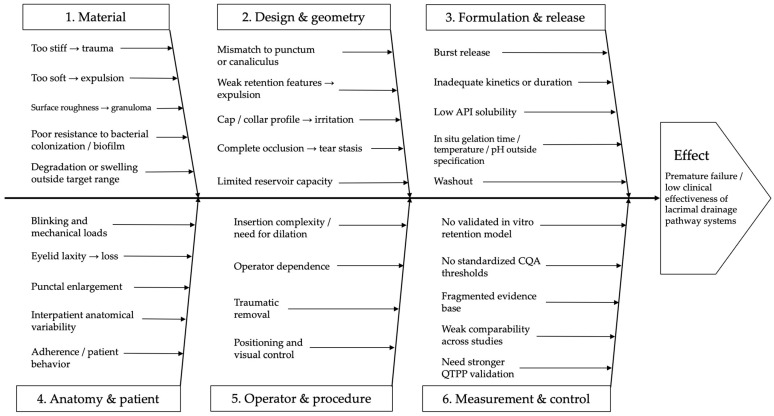
Ishikawa cause-and-effect diagram of QTPP-relevant risks contributing to premature failure or reduced clinical effectiveness of lacrimal drainage pathway drug delivery systems.

To visualize these relationships, an Ishikawa cause-and-effect diagram was constructed (Figure 4). The diagram summarizes the main material-, design-, formulation-, patient-, procedure-, and measurement-related factors that may contribute to premature system failure or reduced clinical effectiveness. This approach emphasizes that successful development of punctal, intracanalicular, drug-eluting, and in situ-forming systems requires not only controlled drug release, but also robust retention, local tissue compatibility, predictable performance, and validated quality-control methods.

The qualitative nature of this diagram relative to a formal quantitative risk-assessment tool (FMEA) is discussed in Section 9.1.

It is evident that for any system intended for long-term use in the lacrimal drainage system, retention in the lacrimal canaliculus will be a critical parameter. At the same time, reliably determining retention qualitatively today is only possible at the in vivo study stage. This leads to many developments, including those described in the above review, failing at the early clinical trial stage.

The lack of instrumental-methodological base for studying this parameter in a laboratory setting can be compensated by creating, validating, and scaling in vitro lacrimal duct models, by analogy with ongoing studies of other dosage forms on nasal cavity models [71], vitreous models [65], and so on.

Identification of critical quality attributes (CQAs) based on the proposed QTPPs should be conducted by making decisions on the degree of influence of the parameters included in the target quality profile on the efficacy and safety of the drug/medical device, as well as the availability of a validated method and reproducible procedure for determining these parameters. The minimum necessary critical parameters for quality assessment and screening of systems placed in the lacrimal drainage system, in our opinion, are presented in Table 9.

Taken together, cross-system analysis reveals several consistent associations between technological characteristics and clinical performance. Retention—the most critical CQA—is primarily determined by device geometry and material properties: L-shaped designs (L-PPDS, N-PPDS) and swelling-based fixation (intracanalicular hydrogels, SmartPlug^®^) achieve higher retention rates than straight-shafted silicone plugs. Systems with insufficient fixation—as observed with OTX-TP, which declined from 100% retention at day 10 to 42% by day 30—demonstrate that even technically sophisticated drug-release platforms fail when retention is compromised, because premature expulsion nullifies any pharmacokinetic advantage. Clinical efficacy further depends on the match between drug loading capacity and disease-specific pharmacodynamic requirements: the lacrimal punctum volume (~0.2–0.5 µL effective reservoir) constrains total drug load to approximately 500 µg, which appears sufficient for postoperative anti-inflammatory therapy (DEXTENZA^®^, 0.4 mg dexamethasone; FDA-approved), but may be suboptimal for IOP-lowering in glaucoma, where daily prostaglandin doses and intraocular pressure response dynamics differ substantially. Patient acceptance is influenced by device visibility, foreign body sensation, and ease of removal—properties governed by material softness, surface smoothness, and fluorescent labelling for position monitoring. These relationships, now summarized in the Ishikawa diagram (Figure 4), indicate that the path to clinical success requires simultaneous optimization of retention, drug loading, and local tolerability rather than sequential attention to each.

Translating these critical attributes into a finished product raises several pharmaceutical-development challenges. Material selection determines both mechanical performance (swelling degree, compressive modulus) and biodegradation behaviour: hydrophilic polymers such as PEG-based crosslinked hydrogels offer predictable biodegradation driven by tear-fluid pH and temperature, whereas non-degradable silicone matrices provide long-term mechanical stability but require device removal. Drug-release behaviour is governed by the interplay between polymer network density, drug solubility, and API–matrix interactions: low-solubility drugs (e.g., latanoprost, dexamethasone, cyclosporine) require careful formulation to avoid excessive burst release, while the small reservoir volume imposes strict constraints on total drug loading. Biocompatibility must be evaluated at both the material level (cytotoxicity, hemolysis, sensitization potential) and the system level (absence of canaliculitis-promoting surface features or bacterial-colonization risk), as clinical data show that even approved materials (silicone, PEG hydrogel) are associated with biofilm formation and granulation tissue in a proportion of patients. Sterilization is a particularly challenging translational step: terminal methods (gamma irradiation, ethylene oxide) may alter polymer crosslinking density, drug stability, and release kinetics, requiring validation studies that are rarely reported in the reviewed preclinical literature. Finally, the translational gap between in vitro and in vivo performance—currently a principal bottleneck of the field—arises because no standardized, validated biorelevant lacrimal-duct model is yet available to predict retention and release before animal or human studies; development of such tools, analogous to USP dissolution models for oral dosage forms, is identified as a priority in our Section Research Perspectives.

## 9. Limitations

### 9.1. Limitations of the Evidence

The evidence base for punctal and intracanalicular drug delivery systems is still limited and uneven. Because peer-reviewed data are scarce, for several systems we deliberately included all available sources, including trial registries, patent documents, regulatory filings, manufacturer materials, and unpublished or secondary reports; such sources carry lower evidentiary weight and were used mainly to describe composition, design, and mechanism rather than to establish efficacy. Some key retention and efficacy figures also originate from older (2007–2013) and predominantly single-centre studies with small samples and are presented descriptively.

To our knowledge, this is the first attempt to translate the fragmented evidence on lacrimal-route delivery systems into explicit, QbD-based QTPP and CQA reference points. We therefore offer them not as validated clinical or regulatory thresholds, but as an initial structured framework that gives developers a concrete starting point and that dedicated preformulation, in vitro, in vivo, and clinical studies can now test, refine, and ultimately validate.

Similarly, the Ishikawa cause-and-effect diagram (Figure 4) is a qualitative, literature-informed risk-mapping tool rather than a formal Failure Mode and Effects Analysis (FMEA). A quantitative FMEA requires reliable Severity, Occurrence, and Detectability scoring for each failure mode; occurrence rates in particular cannot be reliably estimated at present, since most of the reviewed systems have not reached post-market surveillance and lack systematic failure-rate data. We therefore consider the qualitative diagram appropriate for the current stage of evidence synthesis, while acknowledging that quantitative risk prioritization should become feasible as more systems accumulate post-approval clinical experience.

### 9.2. Limitations of the Review Process

As detailed in Section 2, this work is a narrative review and not a systematic review or meta-analysis. The PRISMA-style flow diagram and predefined eligibility criteria were included only to make the literature search transparent and reproducible, and the synthesis was necessarily qualitative; accordingly, the present conclusions are interpretive rather than the product of a quantitative or formally graded evidence assessment. The proposed QTPP/CQA framework should therefore be read as an initial synthesis intended to orient development, not as a definitive standard.

Because this review is intended primarily for developers of pharmaceutical and combination products, and because the reviewed systems differ markedly in development maturity, we grouped them by development stage to help readers weigh the underlying evidence. We adopted a technology-readiness-level (TRL)–type logic for this purpose, while recognising that other classification schemes are equally valid. On this basis, the systems discussed range, for example, from (1) regulatory-approved products (DEXTENZA^®^); through (2) candidates that did not meet their primary Phase III endpoint (OTX-TP); (3) exploratory Phase II programs (OTX-CSI, OTX-DED); (4) preclinical proof-of-concept designs (PEGDA-based 3D-printed and photo-crosslinkable hydrogels); (5) systems characterized only ex vivo or in vitro (thermoreversible poloxamer formulations); to (6) long-marketed occlusive devices supported by relatively sparse comparative data (silicone punctal plugs). The level of evidence, therefore, differs substantially across systems and should be interpreted accordingly.

## 10. Conclusions

This review presents a systematized analysis of medical devices and drug delivery systems utilizing the anatomical features of the lacrimal drainage system—from simple silicone occluders to biodegradable hydrogels and in situ-forming depot matrices. Despite convincing demonstrated potential for sustained release (from 7 days to 3 months) at preclinical and early clinical stages, the translational success and clinical implementation of these developments remain limited.

To date, only one product—DEXTENZA^®^ (intracanalicular dexamethasone insert)—has received FDA approval, while numerous promising platforms (L-PPDS, OTX-TP, OTX-CSI) failed to meet endpoints in late-phase clinical trials due to insufficient efficacy or unexpected safety signals, including canaliculitis (up to 7%) and high extrusion rates.

Based on the reviewed and analyzed experience, several important conclusions were drawn for further scientifically and experimentally substantiated development:

- Retention is at least as critical as drug release. The high rate of premature expulsion (up to 57.4% for some punctal plug designs) nullifies any advantages of the sustained release profile. Mechanical strength and anatomical adaptation (L-shaped design for punctal plugs, swelling capacity for intracanalicular systems) are mandatory critical quality attributes for any system involving this route of administration.
- Drug loading has strict anatomical constraints. The limited volume of the lacrimal punctum (diameter 0.2–0.3 mm) and canalicular lumen (0.5–0.6 mm) restricts total drug loading to approximately 500 µg, which may be insufficient for chronic diseases such as glaucoma. In the case of L-PPDS, limited drug-loading capacity may therefore represent one possible factor contributing to its underperformance; however, inadequate release kinetics at the ocular surface may also have played a role, and the precise mechanism has not been formally established.
- Thermosensitive and photocrosslinkable in situ systems eliminate the size-fitting problem but require strict control of gelation time (≤3 min), phase transition temperature, and pH to prevent premature washout or excessive occlusion. Additional complexity comes from the regulatory classification of such systems (pharmaceutical drug, medical device, or combination product), as well as low system stability and difficulties with scaling such solutions.

The target quality profile (QTPP) matrices and the universal CQA section draft proposed in this review should serve as preformulation guidance. Critical quality attributes, such as swelling degree (30–60%) and burst release control (<40% within the first 24 h) for drug delivery systems, should be routinely determined during development.

Next-generation delivery systems should certainly be oriented toward biodegradable polymers with programmable multi-phase release (e.g., an initial anti-inflammatory ‘burst’ followed by sustained immunosuppression) and built-in imaging labels for non-invasive monitoring of position in the lacrimal drainage system.

Thus, lacrimal drainage pathway delivery systems currently represent a maturing field with one approved product and several discontinued candidates.

The path to success lies not only in achieving sustained release but in ensuring reliable retention, predictable biodegradation, and infection-resistant design—while following QbD principles from the earliest stages of development.

### Research Perspectives

Building on the QTPP/CQA framework proposed in this review, our group intends to investigate this area experimentally, with particular emphasis on in situ-forming systems for intracanalicular administration, which combine minimally invasive, size-independent placement with the potential for sustained, localized drug release. The planned work addresses three interdependent aspects. First, dosage-form design: selection and optimization of the gelation mechanism (thermo-, pH-, or ion-responsive), gelation time and temperature, injectability, and post-gelation rheological strength compatible with the dimensions and physiology of the lacrimal canaliculus. Second, drug-release behaviour: tailoring of release kinetics and duration while limiting burst release, supported by biorelevant in vitro release testing under simulated tear-flow conditions. Third, material selection: identification of biocompatible, biodegradable polymers offering predictable resorption, sufficient mechanical strength, and resistance to bacterial colonization. Across all three aspects, we regard the development, validation, and standardization of in vitro lacrimal-duct models—capable of predicting retention and release before in vivo and clinical evaluation. We hope that the Quality-by-Design-oriented synthesis presented here will serve as a practical starting point for this and similar development efforts.

## Figures and Tables

**Table 1:** Design features and clinical characteristics of permanent punctal plugs.

Plug Studied	Design	Device Characteristics	Results (Adverse Effects, Advantages)
Plug SuperEagle^®^ (EagleVision^®^)	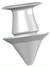	Soft material, thin non-protruding cap. Conical shape and wide curved nose designed to improve device retention [21]	1. Premature expulsion rate 57.4%. 2. Mean time to premature expulsion was 82 days. 3. Granulation tissue formation observed in 34.5% of patients [22]
EaglePlug^®^ (EagleVision^®^)	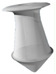	Conical shape aids plug retention. Simple insertion and removal [21]	1. Punctum enlargement observed after premature expulsion. 2. Time to expulsion for this plug type was significantly shorter than that of other devices in the study [23]
EagleFlex Plug^®^ (EagleVision^®^)	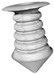	Conical ribbed shaft for improved retention and flexibility, providing 30% more surface area. Thin cap reduces risk of corneal contact [20]	
FCI^®^ plugs: perforated punctal plug, FCI^®^ plug F	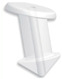	Tilted cap provides better fit, matching natural eyelid anatomy and preventing displacement. Silicone perforated punctal plug coated with hydrophobic PVP (polyvinylpyrrolidone), allowing tear flow through the perforation (description adapted from the manufacturer’s official website).	1. FCI^®^ plug retention rate was 84.2% at three months, 69.5% after one year, and declined to 55.8% after 2 years [24] 2. Six-month retention rate for FCI^®^ plug F was 70.4%. Spontaneous plug loss was associated with larger punctum size in patients [25]
FCI^®^ Ready-Set	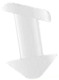	Ultra-thin occluders made from medical-grade silicone. Anatomically shaped to adapt to natural eyelid structure, providing a comfortable fit (description adapted from the manufacturer’s official website).	Biofilm formation and bacterial contamination were detected in 44% of material extracted from plug orifices. Staphylococcus epidermidis was most frequently isolated (75%), followed by Staphylococcus aureus (25%) [26]
SuperFlex^®^ (EagleVision^®^)	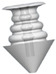	Design based on a flexible ribbed shaft that compresses easily and adapts to lacrimal punctum anatomy. Elastic structure aids reliable retention. Low-profile rim reduces risk of corneal contact, minimizing foreign body sensation after insertion (description adapted from the manufacturer’s official website).	1. Six-month retention rate for SuperFlex^®^ was 32% [27] 2. Spontaneous plug loss was associated with eyelid laxity in elderly patients. Punctum enlargement was also observed [25].
Parasol^®^ Punctal Occluders	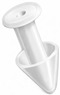	Hollow nose compresses during insertion, in most cases eliminating the need for punctal dilation. Design features a sharp-tipped end and smooth surface facilitating insertion (description adapted from the manufacturer’s official website).	1. Pyogenic granuloma leading to plug extrusion in 4.2% of cases, possibly related to the irregular plug surface causing mucosal damage [21]. 2. Six-month retention rate for Parasol^®^ was 68% [27].

**Table 2:** Design features, materials, and clinical characteristics of intracanalicular plugs.

Plug Studied	Characteristics	Material	Results (Adverse Effects, Advantages)
Collagen plugs	Capable of expanding after insertion; swelling occurs approximately 60% [21]	Bovine collagen	1. Good tolerability; only 3% of the population is allergic to bovine collagen. 2. Granulomatous reaction was observed among adverse effects [21]
SmartPlug^®^	Thermosensitive polymer from which the plug is made changes size and shape after insertion into the lacrimal canaliculus (description adapted from the manufacturer’s official website).	Thermosensitive acrylic material	1. Compared with silicone plug, thermosensitive plug provided greater improvement in ocular surface condition [30] 2. Canaliculitis, dacryocystitis, conjunctivitis, epiphora, and pyogenic granuloma were observed [31,32,33]. 3. Premature expulsion was rare (2.2% of cases) [32].
Herrick Lacrimal Plug^®^	While the collapsible design facilitates insertion, placement within the canaliculus may predispose to tear stasis and secondary infection [21]	Medical-grade silicone	Cases of ocular irritation, epiphora, and intracanalicular plug migration were reported [34].
FORM FIT^®^ plugs	Within 10 min, the hydrogel expands to match the shape of the lacrimal canaliculus, eliminating the need for size matching (description adapted from the manufacturer’s official website).	Hydrogel	Cases of canaliculitis development 6 months after device use and granulation tissue formation 5 years later have also been reported [35].

**Table 3:** Characterization of API delivery systems in the form of punctal plugs. [37,38,39,40,41,42,43,44,45].

Delivery System	Active Substance	Formulation Composition	Estimated Release Duration	Development Stage
L-PPDS	Latanoprost	API polymer matrix, medical-grade silicone, phosphatidylcholine	Approximately 30 days	Phase I
N-PPDS	Nepafenac	API polymer matrix, medical-grade silicone, phosphatidylcholine	No release duration data available	Phase II
EXP-LP	Latanoprost	Composite matrix based on porous carrier material; possible components: silica gel, activated charcoal, zeolite, kaolin/pectin, epoxy or polyurethane matrix, protective coating of parylene or butyral	Up to 3 months	2019 clinical study
EXP-TC	Tacrolimus	Medical-grade composite with tacrolimus; punctal plug design with visible head and central shaft	Up to 3 months	Phase I
3D-printed punctal plugs for controlled ocular delivery	Dexamethasone 10–20%	Irgacure^®^ 819 2%, β-Carotene 1%, PEGDA 61.6–87%, PEG 400 15.4–17.4% (in D10PEG and D20PEG formulations)	4–7 days (depending on formulation)	In vitro testing

**Table 4:** Biodegradable intracanalicular drug delivery systems. [7,50,51,52,53,54,55,56,57,58,59,60,61,62,63].

Biodegradable Intracanalicular Delivery System	Active Substance	Development Stage	Release Duration	Key Results and Limitations
OTX-TP	Travoprost	Phase III	Up to 3 months (~90 days)	In an initial prospective study, IOP reduction reached up to 7.5 mmHg/28%; system retention declined: 100% on day 10 and 42% on day 30. However, Phase III did not demonstrate statistically significant superiority over placebo
OTX-CSI	Cyclosporine	Phase II	3–4 months (~90–120 days)	At 0.36 mg dose and 16-week follow-up, Schirmer test improvement was 1.91–1.98 mm, while the placebo group showed 2.24–3.08 mm, indicating no pronounced advantage
OTX-DED	Dexamethasone	Phase II	2–3 weeks (~14–21 days)	At doses of 0.2 mg/0.3 mg, hyperemia reduction was −0.51/−0.43 compared with −0.21 in the placebo group
DEXTENZA^®^	Dexamethasone	FDA-approved drug	30 days	Absence of pain on day 8—up to 80.4%; absence of anterior chamber cells on day 14—52.3% vs. 31.1% placebo. Adverse effects included: IOP elevation up to 6%, anterior chamber inflammation 10%, reduced visual acuity 2%
Photocrosslinkable hydrogel	Cyclosporine A	In vitro and in vivo	In vitro: 60 days; in vivo: up to 90 days	Swelling increased from 33.9% to 49.5%; residual content on day 60 was CsA > 30%, FK506 > 80%

**Table 5:** In situ-forming intracanalicular systems [65,66,67,69].

In Situ-Forming System	Composition	Selection Criteria	In Vivo and In Vitro Results
Thermosensitive ‘liquid’ hydroxybutyl chitosan plug	Hydroxybutyl chitosan (HBC) solution	Biocompatibility, antibacterial properties, mucoadhesion, solubility, hydrophilicity, non-toxicity, rapid phase transition capability, no size fitting required, reduced infection risk	Phase transition time at body temperature (37 °C) approximately 50 s; effect lasts at least 4 weeks
In situ-forming hydrogel plug	SFMA hydrogel matrix; FTN fluorescent nanoparticles; LAP photoinitiator. Optimized formulation: 8% SFMA, 0.02% FTN, 0.4% LAP	Rapid gelation under visible/blue light; conforming to lacrimal canaliculus shape; biodegradability; non-invasive position monitoring; biocompatibility; temporary lacrimal canaliculus occlusion	In vitro: gelation in 3.3–10.5 s; cytotoxicity not detected up to 800 µg/mL FTN; hemolysis < 3%. In vivo: subcutaneous administration in mice—no pronounced inflammation/fibrosis up to 56 days; insertion into rabbit lacrimal canaliculus—complete occlusion on day 1, partial restoration of permeability within 7 days, therapeutic effect for at least 1 month
Injectable CsA organogel in situ	API—cyclosporine A (CsA); stearic acid, injectable soybean oil, NMP at ratio 1.25:10:0.6 (*w*/*v*/*v*)	Rapid in situ gelation after injection, lacrimal canaliculus occlusion, sustained CsA release	In vitro: reduction in fluid flow to 52.78% at 2 min; in vivo: complete lacrimal pathway occlusion and significant reduction in tear drainage. Bioavailability enhancement: Cmax increased approximately 1.9-fold, AUC 2.15–2.49-fold in ocular tissues compared with eye drops
In situ lacrimal duct implant based on biodegradable thermoreversible polymer formulation	Optimal formulation: poloxamer 407—18.0%, poloxamer 188—3.0%; API—diclofenac 25.0 mg/mL, nitrofuralum at 40 mg per 10 mL polymer base	Preservation of thermoreversible properties, gelation temperature 38.5–40 °C, minimum gelation time, pH compliance with physiological values close to tear fluid pH 7.4	Using a lacrimal duct and sac model at 1:8 scale, gelation temperature of the optimal formulation was 38.7 °C, and gelation time was 150 s

**Table 6:** QTPP for Punctal Plugs.

A. For All Punctal Plugs (Occlusive Devices and Drug Delivery Systems)			
QTPP/Quality Parameter	Optimal/Target Value	How to Control/Method	Basis/Source of Derivation
Reliable retention	Minimal risk of premature expulsion/migration	In clinical studies—assessment of plug retention in the lacrimal punctum	Based on clinical retention/expulsion outcomes reported for commercial punctal plugs and punctal drug delivery systems, including SuperEagle^®^, FCI^®^ plug, SuperFlex^®^, L-PPDS, and N-PPDS [22,24,25,27,39,41]
Size conformance	Diameter matches lacrimal punctum size	Pre-insertion size fitting	Derived from anatomical dimensions of the lacrimal punctum and canaliculus and from dimensions of punctal systems reported in the reviewed studies [12,13,14,38]
Absence of foreign body sensation	Minimal foreign body sensation	Primarily clinical assessment; indirectly controlled by shape, surface smoothness, material softness, and size	Based on clinical tolerability data and design-related factors described for punctal plugs (cap profile, shape, material softness, surface properties) [20,21,27,39]
Surface smoothness	Smooth surface without traumatizing irregularities	Visual inspection, microscopy/SEM	Proposed from reports linking irregular surface or traumatic interaction with irritation and granuloma formation; surface evaluation methods were used in experimental systems [21,28,45]
**B. For punctal Drug Delivery Systems**			
**QTPP/Quality Parameter**	**Optimal/Target Value**	**How to Control/Method**	**Basis/Source of Derivation**
API release duration	Depending on the polymer system and required release profile—from 7 to 60 days	In vitro release profile	Derived from reported release durations: 4–7 days for PEGDA-based 3D-printed plugs, ~30 days for L-PPDS, and up to 3 months for EXP systems [37,38,39,40,41,42,43,44,45]
API loading/drug content	Based on system dimensions, limited to no more than 500 µg	HPLC after extraction	Based on reported loading ranges for punctal systems: L-PPDS (44–81 µg latanoprost) and EXP-LP (250–450 µg latanoprost), interpreted considering device dimensions and anatomical constraints [13,14,39,43]
Biocompatibility/cytocompatibility	Absence of pronounced cytotoxicity and irritation	Cytocompatibility testing	Derived from cytocompatibility results for PEGDA-based systems and clinical safety/tolerability data for punctal delivery systems [21,39,45]

**Table 7:** QTPP for intracanalicular plugs.

A. For All Intracanalicular Plugs (Occlusive Devices and Drug Delivery Systems)			
QTPP/Quality Parameter	Optimal/Target Value	How to Control/Method	Basis/Source of Derivation
Retention in lacrimal canaliculus	At least 80% over 4 weeks	In clinical studies—assessment of plug retention in the lacrimal punctum	Proposed as an expert-derived development target based on reported early retention values for lacrimal systems, including L-PPDS (78% at 4 weeks), N-PPDS (98% at 14 days), and OTX-TP retention profiles reported in different trials [39,41]
Hydrogel swelling degree	Should not be less than 30% and should not exceed 60%	In vitro studies in lacrimal duct model	Derived from reported swelling behavior of collagen plugs (~60%) and photocrosslinked hydrogel systems (33.9–49.5%) [21,63]
Biocompatibility	Absence of cytotoxicity	In vitro: cytocompatibility test using BALB/3T3 fibroblast cell line	Based on preclinical and clinical safety data for hydrogel-based intracanalicular systems [45,63]
Degree of occlusion	Controlled or partial occlusion	In vitro studies in lacrimal duct model	Derived from complication profiles associated with excessive tear stasis, canaliculitis, dacryocystitis, and epiphora in intracanalicular devices [31,32,33,34,35]
Absence of foreign body sensation	Minimal foreign body sensation	Primarily clinical assessment; indirectly controlled by shape, surface smoothness, material softness, and size	Based on tolerability findings reported for intracanalicular systems, including OTX-TP and dexamethasone inserts [53,56,61]
Antimicrobial resistance/infection risk	Material must be resistant to bacterial colonization	In vitro microbiological testing	Derived from reports of bacterial contamination, biofilm formation, canaliculitis, and dacryocystitis in lacrimal devices [26,31,33]
Mechanical strength	Compressive modulus range 91.25–133.4 kPa	Compression testing method	Proposed from the compression testing range reported for photocrosslinked lacrimal duct hydrogel systems [63]
**B. For Intracanalicular Drug Delivery Systems**			
**QTPP/Quality Parameter**	**Optimal/Target Value**	**How to Control/Method**	**Basis/Source of Derivation**
API release	Depending on the polymer system and required release profile—from 7 to 60 days	In vitro/in vivo release profile	Derived from OTX-DED/DEXTENZA^®^-type dexamethasone systems (2–4 weeks) and OTX-CSI/CsA hydrogel systems (up to 3–4 months) [54,55,56,58,63]
API content	0.2–0.4 mg; 10–30%—depending on the system	Quantitative API analysis	Based on reported values for OTX-DED (0.2–0.3 mg dexamethasone), DEXTENZA^®^ (0.4 mg dexamethasone), and experimental CsA/FK506 hydrogels (10–30%) [55,56,63]
Release rate without burst release	No more than 40% released during the first quarter of the application period	In vitro dynamic flow model + HPLC	Proposed as an expert-derived target to avoid excessive burst release; informed by comparison between rapid early release in PEGDA-based systems and more sustained release in hydrogel/organogel platforms [45,63,69]
Matrix biodegradability	Predictable resorption without mandatory removal over the therapeutic action period	Clinical studies, in vivo retention studies	Based on the resorbable behavior of DEXTENZA^®^ and controlled degradation reported for experimental hydrogel systems [56,58,63,67]

**Table 8:** QTPP for in situ-forming systems.

A. For All In Situ-Forming Systems (Occlusion Devices and Drug Delivery Systems)			
QTPP/Quality Parameter	Optimal/Target Value	How to Control/Method	Basis/Source of Derivation
Gelation time	No more than 3 min	Visual gelation time recording/rheology	Derived from reported gelation times: ~50 s for hydroxybutyl chitosan, 3.3–10.5 s for SFMA/FTN hydrogel systems, and ~150 s for the thermoreversible lacrimal implant model [66,67,70]
Gelation temperature	Depending on nosology: up to 37 °C without inflammation, up to 39 °C—with inflammation	Thermostatted lacrimal duct model	Based on reported thermosensitive behavior of hydroxybutyl chitosan and the thermoreversible inflammatory lacrimal implant model (optimal 38.7 °C) [66,70]
System pH	Close to tear fluid pH—approximately 7.4	pH measurement	Derived from formulation selection criteria used for the thermoreversible lacrimal implant [70]
Degree of occlusion	Complete or temporary lacrimal pathway occlusion; fluid flow reduction to 50% within 2 min	Fluid flow model/permeability test	Based on complete temporary occlusion reported for SFMA/FTN hydrogel plugs and fluid flow reduction to 52.78% at 2 min for CsA organogel [67,69]
Swelling and degradation	Controlled biodegradation; partial restoration of permeability within 7 days	In vitro studies in lacrimal duct model	Derived from the SFMA/FTN hydrogel plug model [67]
Rheological properties	Sufficient viscosity/strength after gelation for canalicular retention	Rheological analysis	Based on rheological characterization reported for SFMA/FTN hydrogel and thermoreversible systems [67,70]
Biocompatibility	Absence of pronounced cytotoxicity; hemolysis < 3%; no pronounced inflammation/fibrosis	Cell test, hemolytic test	Derived from cell, hemolysis, and in vivo biocompatibility studies of SFMA/FTN hydrogel plugs [67]
Duration of therapeutic effect	At least 4 weeks	Dynamic efficacy assessment in vitro/in vivo	Based on reported therapeutic duration for hydroxybutyl chitosan and therapeutic/monitoring duration for SFMA/FTN hydrogel systems [66,67]
**B. For In Situ Drug Delivery Systems**			
**QTPP/Quality Parameter**	**Optimal/Target Value**	**How To Control/Method**	**Basis/Source of Derivation**
API release	Sustained API release while maintaining occlusion	In vitro release test	Derived from CsA organogel and related in situ lacrimal drug delivery systems [69,70]
API bioavailability	Target superiority over drops—observed improvement in pharmacokinetic parameters	Pharmacokinetic study	Based on CsA organogel pharmacokinetic data showing ~1.9-fold increase in Cmax and 2.15–2.49-fold increase in AUC compared with eye drops [69]

**Table 9:** Minimum necessary CQAs for medical devices and drug delivery systems in the lacrimal drainage system.

Critical Parameter	Comment
Retention in lacrimal canaliculus	Premature expulsion or device migration nullifies the therapeutic effect. Clinical studies demonstrate a decline in retention from 100% on day 10 to 42% on day 30 (OTX-TP). Target parameter: ≥80% retention over 4 weeks
Occlusion	The degree of lacrimal drainage system blockage determines both the mechanical effect (increased tear residence time) and the risk of complications (epiphora, dacryocystitis). Partial occlusion is preferable to complete occlusion to reduce fluid stasis. Target parameter: fluid flow reduction ≥ 50% within 2 min of introduction
Mechanical strength	The device must withstand compressive loads arising during blinking and eyelid movements without deformation, migration, or fragmentation. Excessively soft materials prematurely extrude; excessively rigid ones traumatize the mucosa. Target parameter: compressive modulus in the range of 91–133 kPa
Swelling	This characteristic is critical for hydrogel systems (intracanalicular plugs, in situ-forming implants). Swelling provides passive fixation without pre-sizing. Target range: 30–60% (based on reported hydrogel/collagen plug data).

## Data Availability

No new data were created or analyzed in this study. Data sharing is not applicable to this article.

## Supplementary Materials

The following supporting information can be downloaded at: https://www.mdpi.com/article/10.3390/pharmaceutics18070830/s1, Table S1: PRISMA 2020 checklist.

## Abbreviations

API	Active pharmaceutical ingredient
AUC	Area under the curve
Cmax	Maximum concentration
CQA	Critical quality attribute
CQAs	Critical quality attributes
CsA	Cyclosporine A
DED	Dry eye disease
DLP	Digital light processing
FDA	Food and Drug Administration
FT-IR	Fourier-transform infrared spectroscopy
FTN	Fluorescent tracing nanoparticles
GRADE	Grading of Recommendations Assessment, Development and Evaluation
HAMA	Methacrylate-modified hyaluronic acid
HBC	Hydroxybutyl chitosan
HPLC	High-performance liquid chromatography
ICH	International Council for Harmonisation
IOP	Intraocular pressure
IVIS	In vivo imaging system
LAP	Lithium phenyl-2,4,6-trimethylbenzoylphosphinate
L-PPDS	Latanoprost punctal plug delivery system
N-PPDS	Nepafenac punctal plug delivery system
NMP	N-methyl-2-pyrrolidone
PEG	Polyethylene glycol
PEG 400	Polyethylene glycol 400
PEGDA	Polyethylene glycol diacrylate
PPDS	Punctal plug delivery system
PRISMA	Preferred Reporting Items for Systematic Reviews and Meta-Analyses
PVP	Polyvinylpyrrolidone
QbD	Quality by design
QTPP	Target product quality profile
SEM	Scanning electron microscopy
SFMA	Silk fibroin methacrylate
TEM	Transmission electron microscopy
UV	Ultraviolet

## References

[1] Lin X., Zhou Y., Lv K., Wu W., Chen C. (2025). Nanomedicine-Based Ophthalmic Drug Delivery Systems for the Treatment of Ocular Diseases. Int. J. Nanomed..

[2] Wang Y., Wang C. (2022). Novel Eye Drop Delivery Systems: Advance on Formulation Design Strategies Targeting Anterior and Posterior Segments of the Eye. Pharmaceutics.

[3] Jumelle C., Gholizadeh S., Annabi N., Dana R. (2020). Advances and Limitations of Drug Delivery Systems Formulated as Eye Drops. J. Control. Release.

[4] Goldstein M.H., Silva F.Q., Blender N., Tran T., Vantipalli S. (2022). Ocular Benzalkonium Chloride Exposure: Problems and Solutions. Eye.

[5] Zaharia A.-C., Dumitrescu O.-M., Radu M., Rogoz R.-E. (2022). Adherence to Therapy in Glaucoma Treatment—A Review. J. Pers. Med..

[6] Ibach M.J., Zimprich L., Wallin D.D., Olevson C., Puls-Boever K., Thompson V. (2022). In Clinic Optometrist Insertion of Dextenza (Dexamethasone Ophthalmic Insert 0.4mg) Prior to Cataract Surgery: The PREPARE Study. Clin. Ophthalmol..

[7] Lee A., Blair H.A. (2020). Dexamethasone Intracanalicular Insert: A Review in Treating Post-Surgical Ocular Pain and Inflammation. Drugs.

[8] Anurova M.N., Bakhrushina E.O., Lapik I.V., Turaeva A.R., Demina N.B., Sysuev B.B., Krasnyuk I.I. (2023). Biopharmaceutical Properties of New Mucoadhesive Dosage Form for Eye Degenerative Diseases Treatment. Drug Dev. Regist..

[9] Weber C., Quintin P., Holz F.G., Fea A., Mercieca K. (2024). Ocular Drug Delivery Systems: Glaucoma Patient Perceptions from a German University Hospital Eye Clinic. Graefes Arch. Clin. Exp. Ophthalmol..

[10] Fea A.M., Vallino V., Cossu M., Marica V., Novarese C., Reibaldi M., Petrillo F. (2024). Drug Delivery Systems for Glaucoma: A Narrative Review. Pharmaceuticals.

[11] Chan H.H., Wong T.T., Lamoureux E., Perera S. (2015). A Survey on the Preference of Sustained Glaucoma Drug Delivery Systems by Singaporean Chinese Patients: A Comparison Between Subconjunctival, Intracameral, and Punctal Plug Routes. J. Glaucoma.

[12] Shkolnik S.F., Shkolnik G.S., Krasnozhen V.N., Zakirova A.M. (2020). Anatomy and Physiology of the Lacrimal Drainage System. Application of New Technologies in the Treatment of Lacrimal Drainage System Pathology.

[13] Maliborski A., Różycki R. (2014). Diagnostic Imaging of the Nasolacrimal Drainage System. Part I. Radiological Anatomy of Lacrimal Pathways. Physiology of Tear Secretion and Tear Outflow. Med. Sci. Monit..

[14] Tyson S.L., Campbell P., Biggins J., Driscoll A., Jarrett P., Gibson A., Vantipalli S., Metzinger J.L., Goldstein M.H. (2020). Punctum and Canalicular Anatomy for Hydrogel-Based Intracanalicular Insert Technology. Ther. Deliv..

[15] Xiao B., Guo D., Liu R., Tu M., Chen Z., Zheng Y., Liu C., Liang L. (2023). Obstruction of the Tear Drainage Altered Lacrimal Gland Structure and Function. Investig. Ophthalmol. Vis. Sci..

[16] Bhujel B., Oh S.-H., Kim C.-M., Yoon Y.-J., Chung H.-S., Ye E.-A., Lee H., Kim J.-Y. (2023). Current Advances in Regenerative Strategies for Dry Eye Diseases: A Comprehensive Review. Bioengineering.

[17] Ervin A., Law A., Pucker A.D. (2017). Punctal Occlusion for Dry Eye Syndrome. Cochrane Database Syst. Rev..

[18] Mansour K., Leonhardt C.J., Kalk W.W., Bootsma H., Bruin K.J., Blanksma L.J., Sjögren Workgroup (2007). Lacrimal Punctum Occlusion in the Treatment of Severe Keratoconjunctivitis Sicca Caused by Sjögren Syndrome: A Uniocular Evaluation. Cornea.

[19] Chen K.-Y., Chan H.-C., Chan C.-M. (2025). How Effective and Safe Are Punctal Plugs in Treating Dry Eye Disease? A Systematic Review and Meta-Analysis. Cont. Lens Anterior Eye.

[20] Jung I., Yoon J.S., Ko B.Y. (2022). Microbiologic Analysis of Removed Silicone Punctal Plugs in Dry Eye Patients. J. Clin. Med..

[21] Jehangir N., Bever G., Mahmood S.M.J., Moshirfar M. (2016). Comprehensive Review of the Literature on Existing Punctal Plugs for the Management of Dry Eye Disease. J. Ophthalmol..

[22] Nasu N., Yokoi N., Nishii M., Komuro A., Inagaki K., Kinoshita S. (2008). Clinical investigation of the extrusion rate and other complications of the new Super Flex Plug punctal plug and other plugs. Nippon. Ganka Gakkai Zasshi.

[23] Sonomura Y., Yokoi N., Komuro A., Inagaki K., Kinoshita S. (2013). Clinical investigation of the extrusion rate and other complications of the SuperEagle plug. Nippon. Ganka Gakkai Zasshi.

[24] Horwath-Winter J., Thaci A., Gruber A., Boldin I. (2007). Long-Term Retention Rates and Complications of Silicone Punctal Plugs in Dry Eye. Am. J. Ophthalmol..

[25] Obata H., Ibaraki N., Tsuru T. (2006). A Technique for Preventing Spontaneous Loss of Lacrimal Punctal Plugs. Am. J. Ophthalmol..

[26] Sugita J., Yokoi N., Fullwood N.J., Quantock A.J., Takada Y., Nakamura Y., Kinoshita S. (2001). The Detection of Bacteria and Bacterial Biofilms in Punctal Plug Holes. Cornea.

[27] Brissette A.R., Mednick Z.D., Schweitzer K.D., Bona M.D., Baxter S.A. (2015). Punctal Plug Retention Rates for the Treatment of Moderate to Severe Dry Eye: A Randomized, Double-Masked, Controlled Clinical Trial. Am. J. Ophthalmol..

[28] Pastor-Pascual F., Aviñó-Martínez J., España-Gregori E., Alcocer-Yuste P. (2007). Pyogenic granuloma following Smart Plug insertion. Arch. Soc. Esp. Oftalmol..

[29] Khanna T., Akkara J.D., Bawa V., Sargunam E.A. (2023). Designing and Making an Open Source, 3D-Printed, Punctal Plug with Drug Delivery System. Indian J. Ophthalmol..

[30] Said A.M.A., Farag M.E., Abdulla T.M., Ziko O.A.O., Osman W.M. (2016). Corneal Sensitivity, Ocular Surface Health and Tear Film Stability after Punctal Plug Therapy of Aqueous Deficient Dry Eye. Int. J. Ophthalmol..

[31] Hill R.H., Norton S.W., Bersani T.A. (2009). Prevalence of Canaliculitis Requiring Removal of SmartPlugs. Ophthalmic Plast. Reconstr. Surg..

[32] Chen S.X., Lee G.A. (2007). SmartPlug in the Management of Severe Dry Eye Syndrome. Cornea.

[33] (2006). SmartPlug Study Group Management of Complications after Insertion of the SmartPlug Punctal Plug: A Study of 28 Patients. Ophthalmology.

[34] Jones C.E., Anklesaria M., Gordon A.D., Prouty R.E., Rashid R., Singla R.K., Schachet J.L. (2002). Retrospective Safety Study of the Herrick Lacrimal Plug: A Device Used to Occlude the Lacrimal Canaliculus. CLAO J..

[35] Joganathan V., Mehta P., Murray A., Durrani O.M. (2010). Complications of Intracanalicular Plugs: A Case Series. Orbit.

[36] Gandhi S., Balas M., Rai A. (2025). Sustainable Solutions for Eye Drop Plastic Waste: Challenges, Innovations, and Environmental Impact. J. Ophthalmol..

[37] Cvenkel B., Kolko M. (2026). Implants to Treat Glaucoma: Promising or Not?. Drugs Aging.

[38] Cadden S., Hao Y., Utkhede D., Rubinchik V., Kjellbotn C.R. (2017). Sustained Release Delivery of Active Agents to Treat Glaucoma and Ocular Hypertension. U.S. Patent.

[39] Kompella U.B., Kadam R.S., Lee V.H. (2010). Recent Advances in Ophthalmic Drug Delivery. Ther. Deliv..

[40] Kompella U.B., Hartman R.R., Patil M.A. (2021). Extraocular, Periocular, and Intraocular Routes for Sustained Drug Delivery for Glaucoma. Prog. Retin. Eye Res..

[41] Mati Therapeutics Inc (2021). Clinical Study Evaluating Safety and Efficacy of a Nepafenac Punctal Plug Delivery System (N-PPDS) Compared with Placebo Punctal Plug Delivery System (p-PPDS) in Controlling Post-Operative Ocular Pain and Inflammation After Routine Unilateral Cataract Surgery. https://clinicaltrials.gov/study/NCT03496467.

[42] Sheetrit E., Halahmi I., Attar I. (2025). Devices and Methods for Delivering a Bio-Active Agent or Bio-Active Agents. U.S. Patent.

[43] Shaare Zedek Medical Center (2017). Safety, Tolerability and Preliminary Efficacy of Unilateral Latanoprost-Loaded Punctual Plug in Patients with Open Angle Early Visual Field Defects Glaucoma or Ocular Hypertension in Comparison to Xalatan© Eye Drops in the Second Eye. https://clinicaltrials.gov/study/NCT03318146.

[44] Eximore Ltd. (2023). Safety, Tolerability, Plug Retention and Preliminary Efficacy of Tacrolimus-Loaded Punctal Plug in Patients with Moderate to Severe Dry Eye Disease—Cohort B. https://clinicaltrials.gov/study/NCT05618730.

[45] Xu X., Awwad S., Diaz-Gomez L., Alvarez-Lorenzo C., Brocchini S., Gaisford S., Goyanes A., Basit A.W. (2021). 3D Printed Punctal Plugs for Controlled Ocular Drug Delivery. Pharmaceutics.

[46] Cooper R.C., Yang H. (2019). Hydrogel-Based Ocular Drug Delivery Systems: Emerging Fabrication Strategies, Applications, and Bench-to-Bedside Manufacturing Considerations. J. Control. Release.

[47] Lynch C.R., Kondiah P.P.D., Choonara Y.E., du Toit L.C., Ally N., Pillay V. (2020). Hydrogel Biomaterials for Application in Ocular Drug Delivery. Front. Bioeng. Biotechnol..

[48] Tsung T.-H., Chen Y.-H., Lu D.-W. (2023). Updates on Biodegradable Formulations for Ocular Drug Delivery. Pharmaceutics.

[49] Sawhney A.S., Jarrett P., Bassett M., Blizzard C. (2013). Drug Delivery Through Hydrogel Plugs. U.S. Patent.

[50] Singh R.B., Ichhpujani P., Thakur S., Jindal S. (2020). Promising Therapeutic Drug Delivery Systems for Glaucoma: A Comprehensive Review. Ther. Adv. Ophthalmol..

[51] Perera S.A., Ting D.S., Nongpiur M.E., Chew P.T., Aquino M.C.D., Sng C.C., Ho S.-W., Aung T. (2016). Feasibility Study of Sustained-Release Travoprost Punctum Plug for Intraocular Pressure Reduction in an Asian Population. Clin. Ophthalmol..

[52] Kesav N.P., Young C.E.C., Ertel M.K., Seibold L.K., Kahook M.Y. (2021). Sustained-Release Drug Delivery Systems for the Treatment of Glaucoma. Int. J. Ophthalmol..

[53] Mohan N., Chakrabarti A., Nazm N., Mehta R., Edward D.P. (2022). Newer Advances in Medical Management of Glaucoma. Indian J. Ophthalmol..

[54] Ocular Therapeutix, Inc. (2023). A Randomized, Multi-Center, Double-Masked, Vehicle-Controlled, Phase 1/2 Study to Evaluate the Safety, Tolerability, and Efficacy of OTX-CSI (Cyclosporine Ophthalmic Insert) for Intracanalicular Use for the Treatment of Subjects with Dry Eye Disease (DED). https://clinicaltrials.gov/study/NCT04362670.

[55] Ocular Therapeutix, Inc. (2023). A Randomized, Double-Masked, Vehicle-Controlled, Phase 2 Study to Evaluate the Efficacy and Safety of OTX-DED (Dexamethasone Intracanalicular Ophthalmic Insert) for the Short-Term Treatment of Signs and Symptoms of Dry Eye Disease (DED). https://clinicaltrials.gov/study/NCT04747977.

[56] U.S. Food and Drug Administration (2025). DEXTENZA (Dexamethasone Ophthalmic Insert) 0.4 Mg, for Intracanalicular Use: Prescribing Information.

[57] McLaurin E.B., Evans D., Repke C.S., Sato M.A., Gomes P.J., Reilly E., Blender N., Silva F.Q., Vantipalli S., Metzinger J.L. (2021). Phase 3 Randomized Study of Efficacy and Safety of a Dexamethasone Intracanalicular Insert in Patients With Allergic Conjunctivitis. Am. J. Ophthalmol..

[58] Blizzard C., McLaurin E.B., Driscoll A., Silva F.Q., Vantipalli S., Metzinger J.L., Goldstein M.H. (2021). Plasma Pharmacokinetic Parameters of Dexamethasone Following Administration of a Dexamethasone Intracanalicular Insert in Healthy Adults. Clin. Ophthalmol..

[59] Walters T., Bafna S. (2016). Efficacy and Safety of Sustained Release Dexamethasone for the Treatment of Ocular Pain and Inflammation after Cataract Surgery: Results from Two Phase 3 Studies. J. Clin. Exp. Ophthalmol..

[60] Tyson S.L., Bafna S., Gira J.P., Goldberg D.F., Jones J.J., Jones M.P., Kim J.K., Martel J.M., Nordlund M.L., Piovanetti-Perez I.K. (2019). Multicenter Randomized Phase 3 Study of a Sustained-Release Intracanalicular Dexamethasone Insert for Treatment of Ocular Inflammation and Pain after Cataract Surgery. J. Cataract. Refract. Surg..

[61] Nijm L., Matossian C., Rhee M.K., Stephens J.D., Rosselson M.E., Majmudar P.A., Gollamudi S.R., Patel R.H., Bauskar A., Montieth A. (2024). Early Real-World Patient and Staff Experience with an Intracanalicular Dexamethasone Insert. Clin. Ophthalmol..

[62] Lin T., Wang W., Chen T., Bao B., Liu T., Zhao H., Feng C., Lin Q., Zhu L., Gong L. (2023). A Lacrimal Duct Drug Delivery System Based on Photo-Induced Hydrogel for Dry Eye and Allergic Conjunctivitis Therapy. Compos. Part B Eng..

[63] Zhang Q., Yan K., Zheng X., Liu Q., Han Y., Liu Z. (2024). Research Progress of Photo-Crosslink Hydrogels in Ophthalmology: A Comprehensive Review Focus on the Applications. Mater. Today Bio.

[64] Ahmed B., Jaiswal S., Naryal S., Shah R.M., Alany R.G., Kaur I.P. (2024). In Situ Gelling Systems for Ocular Drug Delivery. J. Control. Release.

[65] Bakhrushina E.O., Dubova A.I., Nikonenko M.S., Grikh V.V., Shumkova M.M., Korochkina T.V., Krasnyuk I.I., Krasnyuk I.I. (2023). Thermosensitive Intravitreal In Situ Implant of Cefuroxime Based on Poloxamer 407 and Hyaluronic Acid. Gels.

[66] Lin T., Wang W., Lu Y., Gong L. (2021). Treatment of Dry Eye With Intracanalicular Injection of Hydroxybutyl Chitosan: A Prospective Randomized Clinical Trial. Front. Med..

[67] Dai M., Xu K., Xiao D., Zheng Y., Zheng Q., Shen J., Qian Y., Chen W. (2022). In Situ Forming Hydrogel as a Tracer and Degradable Lacrimal Plug for Dry Eye Treatment. Adv. Healthc. Mater..

[68] Raut S., Azheruddin M., Kumar R., Singh S., Giram P.S., Datta D. (2024). Lecithin Organogel: A Promising Carrier for the Treatment of Skin Diseases. ACS Omega.

[69] Cao Z., Chen Y., Bai S., Zheng Z., Liu Y., Gui S., Shan S., Wu J., He N. (2023). In Situ Formation of Injectable Organogels for Punctal Occlusion and Sustained Release of Therapeutics: Design, Preparation, in Vitro and in Vivo Evaluation. Int. J. Pharm..

[70] Bakhrushina E.O., Ermachkova M.A., Dubova A.I., Demina N.B., Krasnyuk I.I. (2022). Development of an in situ implant for the treatment of inflammatory diseases of the lacrimal duct using in vitro modeling techniques. Proc. Voronezh State Univ. Ser. Chem. Biol. Pharm..

[71] Mikhel I., Bakhrushina E., Abuelez S., Eremeeva K., Yang X., Svistushkin V., Stepanova O.I., Krasnyuk I.I., Zhemerikin G., Krasnyuk I.I. (2025). 3D PRINTING TECHNOLOGIES IN THE DEVELOPMENT OF A BIORELEVANT IN VITRO MODEL OF THE NASAL CAVITY: NEW STEP OF INTRANASAL DRUGS QUALITY ASSESSMENT. Int. J. Appl. Pharm..

