# Supplementary material for: Punctal and Intracanalicular Drug Delivery Systems for Ophthalmic Use: A Narrative Review of Technologies, Clinical Outcomes, and Critical Quality Attributes
Source: Pharmaceutics. 2026 Jul 7;18(7):830. doi: 10.3390/pharmaceutics18070830 (PMC13414552; doi:10.3390/pharmaceutics18070830)
Supplement: Supplementary file 1 [file pharmaceutics-18-00830-s001.zip › pharmaceutics-4406389-supplementary.pdf]

PRISMA 2020 Checklist

Section and Topic	Item #	Checklist item	Location where item is reported
**TITLE**			
Title	1	Identify the report as a systematic review.	Title page. The manuscript is identified as a narrative review rather than a systematic review.
**ABSTRACT**			
Abstract	2	See the PRISMA 2020 for Abstracts checklist.	The abstract includes Background, Objective, Methods, Results, and Conclusion.
**INTRODUCTION**			
Rationale	3	Describe the rationale for the review in the context of existing knowledge.	Introduction, paragraphs describing limitations of conventional eye drops, patient adherence problems, and the rationale for sustained-release lacrimal drainage system delivery.
Objectives	4	Provide an explicit statement of the objective(s) or question(s) the review addresses.	Introduction, final paragraph.
**METHODS**			
Eligibility criteria	5	Specify the inclusion and exclusion criteria for the review and how studies were grouped for the syntheses.	Section 2.2. Inclusion and Exclusion Criteria; Section 2.4. Data Extraction and Synthesis.
Information sources	6	Specify all databases, registers, websites, organisations, reference lists and other sources searched or consulted to identify studies. Specify the date when each source was last searched or consulted.	Section 2.1. Literature Search Strategy; Section 2.3. Source Selection and Systematization. Note: the date of the last search should be added to Section 2.1 if required by the journal.
Search strategy	7	Present the full search strategies for all databases, registers and websites, including any filters and limits used.	Section 2.1. Literature Search Strategy. Search keywords and Boolean operators are reported. Full database-specific search strings are not provided; if added, cite Supplementary Table S1.
Selection process	8	Specify the methods used to decide whether a study met the inclusion criteria of the review, including how many reviewers screened each record and each report retrieved, whether they worked independently, and if applicable, details of automation tools used in the process.	Section 2.3. Source Selection and Systematization; Figure 1. The number of records excluded by automated and manual screening is reported. The number of reviewers and whether screening was independent are not explicitly reported.
Data collection process	9	Specify the methods used to collect data from reports, including how many reviewers collected data from each report, whether they worked independently, any processes for obtaining or confirming data from study investigators, and if applicable, details of automation tools used in the process.	Section 2.4. Data Extraction and Synthesis. Extracted data categories are described. The number of reviewers involved in data extraction is not explicitly reported.
Data items	10a	List and define all outcomes for which data were sought. Specify whether all results that were compatible with each outcome domain in each study were sought (e.g. for all measures, time points, analyses), and if not, the methods	Section 2.4. Data Extraction and Synthesis. Clinical and technological

Section and Topic	Item #	Checklist item	Location where item is reported
		used to decide which results to collect.	data items included efficacy endpoints, retention rate, safety profile, complications, and release duration.
	10b	List and define all other variables for which data were sought (e.g. participant and intervention characteristics, funding sources). Describe any assumptions made about any missing or unclear information.	Section 2.4. Data Extraction and Synthesis. Other extracted variables included system type, device location, material/polymer matrix, active substance, retention mechanism, occlusion or phase transition mechanism, development stage, study design, and limitations.
Study risk of bias assessment	11	Specify the methods used to assess risk of bias in the included studies, including details of the tool(s) used, how many reviewers assessed each study and whether they worked independently, and if applicable, details of automation tools used in the process.	Section 2.5. Evidence Level Assessment. Formal risk-of-bias assessment was not performed because of the narrative nature of the review and heterogeneity of included sources.
Effect measures	12	Specify for each outcome the effect measure(s) (e.g. risk ratio, mean difference) used in the synthesis or presentation of results.	Not applicable. No meta-analysis or predefined quantitative effect-measure synthesis was performed. Results were presented narratively.
Synthesis methods	13a	Describe the processes used to decide which studies were eligible for each synthesis (e.g. tabulating the study intervention characteristics and comparing against the planned groups for each synthesis (item #5)).	Section 2.4. Data Extraction and Synthesis; Section 2.6. Data Synthesis and Substantiation of Critical Quality Attributes. Sources were grouped by thematic sections and system type.
	13b	Describe any methods required to prepare the data for presentation or synthesis, such as handling of missing summary statistics, or data conversions.	Section 2.4. Data Extraction and Synthesis. No quantitative data conversions or handling of missing summary statistics were required because the synthesis was qualitative.
	13c	Describe any methods used to tabulate or visually display results of individual studies and syntheses.	Section 2.4. Data Extraction and Synthesis; Tables 1–9; Figures 1–4
	13d	Describe any methods used to synthesize results and provide a rationale for the choice(s). If meta-analysis was performed, describe the model(s), method(s) to identify the presence and extent of statistical heterogeneity, and software package(s) used.	Section 2.4. Data Extraction and Synthesis; Section 2.6. Data Synthesis and Substantiation of Critical Quality Attributes. Data were synthesized qualitatively; meta-analysis was not performed.
	13e	Describe any methods used to explore possible causes of heterogeneity among study results (e.g. subgroup analysis, meta-regression).	Not applicable. Subgroup analysis, meta-regression, and formal exploration of statistical heterogeneity were not performed.
	13f	Describe any sensitivity analyses conducted to assess robustness of the synthesized results.	Not applicable. Sensitivity analyses were

Section and Topic	Item #	Checklist item	Location where item is reported
			not performed.
Reporting bias assessment	14	Describe any methods used to assess risk of bias due to missing results in a synthesis (arising from reporting biases).	Not applicable. Reporting bias due to missing results was not formally assessed because no statistical synthesis/meta-analysis was performed.
Certainty assessment	15	Describe any methods used to assess certainty (or confidence) in the body of evidence for an outcome.	Section 2.5. Evidence Level Assessment. Formal certainty-of-evidence assessment using GRADE or similar tools was not performed.
**RESULTS**			
Study selection	16a	Describe the results of the search and selection process, from the number of records identified in the search to the number of studies included in the review, ideally using a flow diagram.	Section 2.3. Source Selection and Systematization; Figure 1. PRISMA flow diagram.
	16b	Cite studies that might appear to meet the inclusion criteria, but which were excluded, and explain why they were excluded.	Section 2.3. Source Selection and Systematization. Reasons for exclusion after full-text assessment are reported. Individual excluded studies are not listed; if added, cite Supplementary Table S2.
Study characteristics	17	Cite each included study and present its characteristics.	Sections 3–8 and Tables 1–8. Characteristics of included systems and studies are presented by thematic group: occlusive devices, punctal drug delivery systems, intracanalicular systems, in situ-forming systems, and QTPP/CQA matrices.
Risk of bias in studies	18	Present assessments of risk of bias for each included study.	Section 2.5. Evidence Level Assessment. Formal study-level risk-of-bias assessment was not performed.
Results of individual studies	19	For all outcomes, present, for each study: (a) summary statistics for each group (where appropriate) and (b) an effect estimate and its precision (e.g. confidence/credible interval), ideally using structured tables or plots.	Sections 4–7 and Tables 1–5. Results of individual studies and systems are presented narratively, including retention, safety, release duration, efficacy findings, and limitations where available.
Results of syntheses	20a	For each synthesis, briefly summarise the characteristics and risk of bias among contributing studies.	Not applicable. No formal statistical synthesis was conducted. Characteristics and evidence level considerations are discussed narratively in Sections 2.5 and 4–8.
	20b	Present results of all statistical syntheses conducted. If meta-analysis was done, present for each the summary estimate and its precision (e.g. confidence/credible interval) and measures of statistical heterogeneity. If comparing groups, describe the direction of the effect.	Not applicable. No meta-analysis or statistical synthesis was performed.

PRISMA 2020 Checklist

Section and Topic	Item #	Checklist item	Location where item is reported
	20c	Present results of all investigations of possible causes of heterogeneity among study results.	Not applicable. Formal investigations of causes of heterogeneity were not performed.
	20d	Present results of all sensitivity analyses conducted to assess the robustness of the synthesized results.	Not applicable. Sensitivity analyses were not performed.
Reporting biases	21	Present assessments of risk of bias due to missing results (arising from reporting biases) for each synthesis assessed.	Not applicable. Reporting bias due to missing results was not formally assessed.
Certainty of evidence	22	Present assessments of certainty (or confidence) in the body of evidence for each outcome assessed.	Section 2.5. Evidence Level Assessment. Formal certainty-of-evidence assessment was not performed.
**DISCUSSION**			
Discussion	23a	Provide a general interpretation of the results in the context of other evidence.	Sections 4–8 and Conclusions. Results are interpreted in the context of available clinical, technological, regulatory, patent, and preclinical evidence.
	23b	Discuss any limitations of the evidence included in the review.	Sections 2.5, 4–8, Conclusions, Section 9.1 (Limitations of the Evidence). Limitations of the evidence are discussed throughout the review, including heterogeneity of sources, unpublished clinical trial results, and limited representative quantitative data.
	23c	Discuss any limitations of the review processes used.	Section 9.2 (Limitations of the Review Process). Limitations of the review process include narrative design, heterogeneity of included sources, and absence of formal risk-of-bias, certainty-of-evidence, and meta-analytic assessment.
	23d	Discuss implications of the results for practice, policy, and future research.	Section 8. Target Product Quality Profile for Development of Products for Lacrimal Drainage System Placement; Conclusions. Implications for product development, QTPP/CQA justification, and future research are discussed.
**OTHER INFORMATION**			
Registration and protocol	24a	Provide registration information for the review, including register name and registration number, or state that the review was not registered.	Section 2.3. Source Selection and Systematization. The review was not registered in PROSPERO or any other systematic review registry.

PRISMA 2020 Checklist

Section and Topic	Item #	Checklist item	Location where item is reported
	24b	Indicate where the review protocol can be accessed, or state that a protocol was not prepared.	Section 2.3. Source Selection and Systematization. A separate review protocol was not prepared.
	24c	Describe and explain any amendments to information provided at registration or in the protocol.	Not applicable. The review was not registered and no protocol was prepared; therefore, protocol amendments were not applicable.
Support	25	Describe sources of financial or non-financial support for the review, and the role of the funders or sponsors in the review.	Funding section. This research received no external funding.
Competing interests	26	Declare any competing interests of review authors.	Conflicts of Interest section. The authors declare no conflicts of interest.
Availability of data, code and other materials	27	Report which of the following are publicly available and where they can be found: template data collection forms; data extracted from included studies; data used for all analyses; analytic code; any other materials used in the review.	Data Availability Statement. No new data were created or analyzed in this study; data sharing is not applicable. No analytic code was used.

From: Page MJ, McKenzie JE, Bossuyt PM, Boutron I, Hoffmann TC, Mulrow CD, et al. The PRISMA 2020 statement: an updated guideline for reporting systematic reviews. BMJ 2021;372:n71. doi: 10.1136/bmj.n71. This work is licensed under CC BY 4.0. To view a copy of this license, visit https://creativecommons.org/licenses/by/4.0/
